# Discovery of a Siderophore Export System Essential for Virulence of *Mycobacterium tuberculosis*

**DOI:** 10.1371/journal.ppat.1003120

**Published:** 2013-01-31

**Authors:** Ryan M. Wells, Christopher M. Jones, Zhaoyong Xi, Alexander Speer, Olga Danilchanka, Kathryn S. Doornbos, Peibei Sun, Fangming Wu, Changlin Tian, Michael Niederweis

**Affiliations:** 1 Department of Microbiology, University of Alabama at Birmingham, Birmingham, Alabama, United States of America; 2 School of Chemistry and Material Sciences, University of Science and Technology of China, Hefei, P. R. China; 3 School of Life Sciences, University of Science and Technology of China, Hefei, P. R. China; 4 High Magnetic Field Laboratory, Chinese Academy of Sciences, Hefei, P. R. China; Johns Hopkins School of Medicine, United States of America

## Abstract

Iron is an essential nutrient for most bacterial pathogens, but is restricted by the host immune system. *Mycobacterium tuberculosis* (*Mtb*) utilizes two classes of small molecules, mycobactins and carboxymycobactins, to capture iron from the human host. Here, we show that an *Mtb* mutant lacking the *mmpS4* and *mmpS5* genes did not grow under low iron conditions. A cytoplasmic iron reporter indicated that the double mutant experienced iron starvation even under high-iron conditions. Loss of *mmpS4* and *mmpS5* did not change uptake of carboxymycobactin by *Mtb*. Thin layer chromatography showed that the Δ*mmpS4/S5* mutant was strongly impaired in biosynthesis and secretion of siderophores. Pull-down experiments with purified proteins demonstrated that MmpS4 binds to a periplasmic loop of the associated transporter protein MmpL4. This interaction was corroborated by genetic experiments. While MmpS5 interacted only with MmpL5, MmpS4 interacted with both MmpL4 and MmpL5. These results identified MmpS4/MmpL4 and MmpS5/MmpL5 as siderophore export systems in *Mtb* and revealed that the MmpL proteins transport small molecules other than lipids. MmpS4 and MmpS5 resemble periplasmic adapter proteins of tripartite efflux pumps of Gram-negative bacteria, however, they are not only required for export but also for efficient siderophore synthesis. Membrane association of MbtG suggests a link between siderophore synthesis and transport. The structure of the soluble domain of MmpS4 (residues 52–140) was solved by NMR and indicates that mycobacterial MmpS proteins constitute a novel class of transport accessory proteins. The bacterial burden of the *mmpS4/S5* deletion mutant in mouse lungs was lower by 10,000-fold and none of the infected mice died within 180 days compared to wild-type *Mtb*. This is the strongest attenuation observed so far for *Mtb* mutants lacking genes involved in iron utilization. In conclusion, this study identified the first components of novel siderophore export systems which are essential for virulence of *Mtb*.

## Author Summary

In the late 19^th^ century the French physician Armand Trousseau recognized that treating anemic tuberculosis patients with iron salts exacerbated the disease. In 1911 Twort postulated that mycobacteria produce an essential growth factor which was identified in 1953 as mycobactin. The hydrophobic mycobactin and its more water-soluble cousin carboxymycobactin are small molecules made by *Mycobacterium tuberculosis* to scavenge iron from its human host. While the biosynthesis of these siderophores has been decoded, it was unknown how *M. tuberculosis* secretes these molecules. In this study, we identified two similar transport systems, MmpS4/MmpL4 and MmpS5/MmpL5, which are required for biosynthesis and export of siderophores by *M. tuberculosis*. The lack of these transport systems drastically decreased the number of *M. tuberculosis* cells in the lungs and spleens of infected mice. Lung examination and histological assessment in mice infected with the *mmpS4/S5* deletion strain showed almost no signs of infection. Further, none of the mice infected with this strain died within 180 days in contrast to wild-type *M. tuberculosis*. In this study, we identified the first components of a novel siderophore export system in *M. tuberculosis* and showed the importance of siderophore export for virulence of *M. tuberculosis*.

## Introduction

Iron is an essential micronutrient for most forms of life on earth because of its vital role as a redox cofactor of proteins required for critical cellular processes. Pathogenic bacteria have evolved an array of intricate mechanisms to scavenge limited iron from the host [1]. *Mycobacterium tuberculosis* (*Mtb*), one of the most successful human bacterial pathogens, is no exception. *Mtb* meets its iron demands by stripping host iron stores employing two hydroxyphenyloxazoline siderophores, mycobactin (MBT) and carboxymycobactin (cMBT). To counteract these bacterial iron acquisition processes, the alveolar macrophage in which *Mtb* thrives, keeps phagosomal iron levels extremely low by the natural resistance-associated macrophage protein Nramp1 in particular after activation by interferon-γ [2], [3]. MBT and cMBT increase the biologically available iron within the phagosomal compartment almost by 20-fold indicating that the *Mtb* siderophores can overcome these host defense mechanisms [4]. Furthermore, studies using siderophore biosynthesis and uptake mutants underpin the importance of siderophore-mediated iron acquisition to the virulence of *Mtb* [5], [6], [7].

In *Mtb*, siderophore biosynthesis is induced under low-iron conditions. When sufficient iron is available the regulator IdeR represses expression of MBT biosynthesis genes *mbtA-N*. The inner membrane transporter IrtAB and the Esx-3 secretion machinery are required for utilization and uptake of siderophores [6], [7], [8]. In *M. smegmatis*, export of the siderophore exochelin was shown to be mediated by ABC-like exporter ExiT [9]. Given mycobacteria's unique outer membrane [10], it is likely that a siderophore secretion system of *Mtb* requires both inner and outer membrane components [11], similarly to the EntS-TolC system of *E. coli* [12], [13].

In this study, we examined two iron-regulated genes encoding predicted outer membrane proteins MmpS4 and MmpS5. We show that either MmpS4 or MmpS5 is required for growth of *Mtb* under low iron conditions. While single *mmpS4* or *mmpS5* deletion mutants do not exhibit a low iron growth phenotype, they have diminished virulence compared to the wild-type strain. Deletion of both *mmpS4* and *mmpS5* drastically decreases synthesis and secretion of siderophores in *Mtb* and greatly reduces its virulence in mice. Subcellular fractionation reveals that MmpS4 and MmpS5 are membrane associated. This study identifies MmpS4 and MmpS5 as the first components of a novel siderophore export system that is crucial for survival of *Mtb* in its host.

## Results

### MmpS4 or MmpS5 is required for growth of *M. tuberculosis* under iron-limited conditions

**Figure 1:**
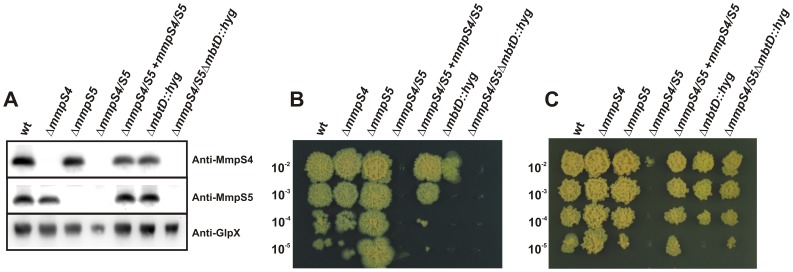
MmpS4 or MmpS5 is required for growth of *M. tuberculosis* under iron-limited conditions. **A.** Expression of MmpS4 and MmpS5 in *Mtb*. Whole cell lysates from wt *Mtb* mc^2^6230, Δ*mmpS4*, Δ*mmpS5*, Δ*mmpS4/S5*, Δ*mmpS4/S5+mmpS4/S5*, Δ*mbtD*::*hyg*, and Δ*mmpS4/S5/mbtD*::*hyg* were probed by Western blot by using rabbit polyclonal antibodies raised against MmpS4 and MmpS5. The cytoplasmic fructose 1,6-bisphosphatase GlpX was used as a loading control and detected using an anti-GlpX antiserum. **B, C.** Serial dilutions of log-phase cultures of *Mtb* mc^2^6230, Δ*mmpS4*, Δ*mmpS5*, Δ*mmpS4/S5*, Δ*mmpS4/S5* fully complemented with *mmpS4* and *mmpS5*, Δ*mbtD*::*hyg*, and Δ*mmpS4/S5*Δ*mbtD*::*hyg* were spotted on low iron glycerol-alanine-salts (GAS) plates (**B**), or on low iron GAS plates with 5 µM hemoglobin as an iron source (**C**).

To investigate whether MmpS4 and MmpS5 are important for growth under iron-deplete conditions, mutants with in-frame deletions of the *mmpS4* and *mmpS5* genes were constructed using homologous recombination in both virulent *Mtb* H37Rv and avirulent *Mtb* mc^2^6230 (ΔRD1, Δ*panCD*; Table S1). Since no low-iron growth defect was observed with the single Δ*mmpS4* and Δ*mmpS5* mutants (Figs. 1, S1–2), and expression of both *mmpS4* and *mmpS5* is induced under iron-limited conditions [14] we suspected that they might have redundant functions. Therefore, we constructed a double *mmpS4/mmpS5* mutant using the single Δ*mmpS5* mutant as the parent strain in both virulent and avirulent *Mtb*. The mutant strains were unmarked by site-specific recombination and confirmed by Southern blot analysis (Fig. S3). Western blot experiments demonstrated the absence of MmpS4 and MmpS5 in the double mutant and in the respective single mutants, while wild-type levels of both proteins were observed in the complemented strains (Figs. 1A, S4). No differences in growth of the single Δ*mmpS4* and Δ*mmpS5* strains were observed on self-made low iron glycerol-alanine salts (GAS) agar plates (Fig. 1B). By contrast, the Δ*mmpS4/S5* mutant did not grow on GAS agar plates (Fig. 1B). Growth of the double mutant was partially rescued when GAS agar plates were supplemented with 5 µM hemoglobin that was previously shown to function as an iron source for *Mtb* (Fig. 1C) [15]. However, in liquid medium, the addition of hemin completely rescued the growth of the Δ*mmpS4/S5* mutant (Fig. S1) verifying that the growth defect of this strain is indeed iron dependent. Complementation of the Δ*mmpS4/S5* mutant with *mmpS4* and *mmpS5* restored growth on low iron plates to wt levels (Fig. 1B). Interestingly, blocking siderophore biosynthesis by insertion of a resistance cassette into *mbtD* in the Δ*mmpS4/S5* double deletion mutant (Δ*mmpS4/S5/*Δ*mbtD::hyg* strain) also restored growth on hemoglobin plates to the level of the wt strain (Fig. 1C). These results indicate that siderophore biosynthesis impairs the growth of this mutant despite the availability of an alternative iron source.

To provide further evidence for the iron growth defect of the Δ*mmpS4/S5* double mutant, growth experiments were conducted in various low iron conditions that included self-made low iron 7H9 medium, or the addition of 2,2′-dipyridyl (DIP) or desferrioxamine (DFO) as ferrous and ferric specific chelators, respectively, to standard 7H9 medium (Figs. S1–2, S5–6). Under each low iron growth condition, Δ*mmpS4/S5* failed to grow. Unlike on solid media, in iron-replete liquid media, Δ*mmpS4/S5* had only a slightly delayed growth phenotype and eventually reached optical densities equal to the wt strain. It is concluded that deletion of *mmpS4* and *mmpS5* confers a low-iron growth defect phenotype in *Mtb*.

### MmpS4 and MmpS5 are not involved in sensing or uptake of iron

**Figure 2:**
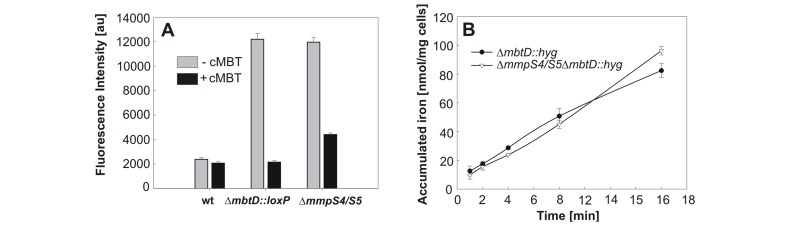
MmpS4 and MmpS5 are not involved in iron sensing or uptake of siderophores. **A.** GFP fluorescence was measured in wt *Mtb* mc^2^6230, Δ*mmpS4/S5*, and Δ*mbtD*::*loxP* strains containing a *gfp*-based iron-regulated reporter construct. Strains were grown in 7H9 media and fluorescence was measured two days after the addition of carboxymycobactin (cMBT) (black bars) or blank control (grey bars). Experiments were performed in triplicate and are shown with standard deviations. **B.** Uptake of ^55^Fe loaded cMBT by *Mtb* Δ*mmpS4/S5* (black circles) and Δ*mmpS4/S5* Δ*mbtD::hyg* (white triangles). Assays were performed at 37°C using a final concentration of 0.25 µM cMBT, 0.45 µCi ^55^Fe in triplicate. Standard deviations are shown.

The inability of *Mtb* Δ*mmpS4/S5* to grow under iron-limiting conditions may be due to defects in siderophore biosynthesis, iron sensing, uptake or secretion of siderophores. Recently, a biosynthetic pathway has been proposed based on the substrate specificities of enzymes encoded by the *mbt* gene cluster [16] which accounts for all enzymatic activities required for MBT biosynthesis. Therefore, it is unlikely that MmpS4 and MmpS5 play a direct role in biosynthesis of MBT and cMBT. To test whether the ability of the Δ*mmpS4/S5* mutant is impaired in sensing low iron conditions, we utilized a *gfp*-based iron-regulated reporter construct [17]. Under low iron conditions, transcription from IdeR-regulated promoters was induced in wt *Mtb* containing the reporter construct as indicated by a strongly increased GFP fluorescence (Fig. S7). However, when wt *Mtb* was grown under high iron conditions, only background fluorescence was observed confirming that *Mtb* senses iron availability (Fig. 2A). To examine the iron sensing capability of the Δ*mmpS4/S5* mutant we exploited the observation that removal of the antibiotic resistance cassette from the MBT biosynthesis mutant ML1600 (Δ*mbtD*::*hyg*) [18] resulted in the strain ML1610 (Δ*mbtD*::*loxP*) (Table S1) with only a partial low-iron growth defect *in vitro* (Fig. S8). This result suggests that replacing *mbtD* with the *hyg* cassette inhibits expression of downstream genes thereby completely eliminating siderophore production. IdeR-regulated promoters are induced under high-iron conditions in *Mtb* Δ*mbtD*::*loxP*, but the addition of exogenous cMBT to this mutant repressed these promoters, demonstrating that this mutant is capable of sensing iron availability and is suitable as a control strain (Fig. 2A). Likewise, IdeR-regulated promoters were induced in the Δ*mmpS4/S5* mutant under high iron conditions, but were repressed after addition of cMBT (Fig. 2A), demonstrating that the Δ*mmpS4/S5* mutant is capable of sensing iron availability.

To test whether MmpS4 and MmpS5 are involved in siderophore uptake we monitored the accumulation of ^55^Fe-loaded cMBT. Iron-loaded cMBT has been shown to donate its iron to MBT in the cell envelope of *Mtb* in addition to being taken up via the inner membrane ABC-transporter IrtAB [8], [19]. To rule out the possibility that differences in MBT levels affected the measured iron uptake rates, we examined ^55^Fe-cMBT uptake at 37°C in the siderophore biosynthesis mutant Δ*mbtD*::*hyg* and the triple mutant Δ*mmpS4*/*S5*/Δ*mbtD*::*hyg* (Fig. 2B). Despite the absence of MBTs/cMTBs no differences were observed in the amount of iron accumulated by these strains. Another control experiment showed that only background ^55^Fe levels were associated with cells at 4°C, indicating that the cell-associated ^55^Fe observed at 37°C was indeed transported inside the cell and not adsorbed at the cell surface (not shown). Taken together, these results demonstrate that MmpS4 and MmpS5 are not involved in uptake of cMBT.

### MmpS4 and MmpS5 are required for siderophore biosynthesis and export by *M. tuberculosis*

**Figure 3:**
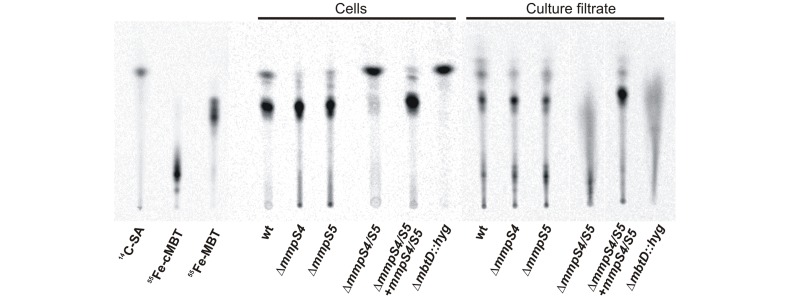
MmpS4 and MmpS5 are required for siderophore secretion in *M. tuberculosis*. TLC of cell-associated and secreted siderophores extracted from cultures of wt *Mtb* H37Rv parent strain ML617, Δ*mmpS4* single deletion mutant ML472, Δ*mmpS5* single deletion mutant ML405, *mmpS4/S5* double deletion mutant ML618, Δ*mmpS4/S5* double deletion mutant fully complemented with *mmpS4* and *mmpS5* ML624, and the siderophore biosynthetic mutant Δ*mbtD*::*hyg* ML1424. Cultures were labelled with 7-[^14^C]-salicylic acid, which was run on the TLC as a control alongside ^55^Fe-loaded cMBT and mycobactin (MBT). Lanes containing cell-associated extracts were loaded with 5,000 cpm, while media extracts were loaded with 7,500 cpm.

The low iron growth defect of the Δ*mmpS4/S5* mutant is not caused by an iron sensing defect or by lack of cMBT uptake. An alternative explanation might be a defect in secretion of cMBT. To this end, cMBTs in wt *Mtb* and in the Δ*mmpS4/S5* mutant were radioactively labeled by feeding the bacteria the biosynthetic precursor 7-[^14^C]-salicylic acid. Cell-associated and secreted siderophores were extracted using chloroform from cell pellets and from the culture filtrate and analyzed by thin-layer chromatography (TLC). As controls, purified and deferrated MBTs/cMBTs from *M. bovis* BCG were loaded with ^55^Fe and used to visualize siderophore spots. TLC analysis demonstrated that purified siderophores from *M. bovis* BCG had the same *R*_f_ values as siderophores from *Mtb* validating their use as controls (Fig. 3). The extracts of cell pellets and of culture supernatants showed that the single deletion mutants *Mtb* Δ*mmpS4* and Δ*mmpS5* synthesized and secreted siderophores as wt *Mtb* (Fig. 3). By contrast, the double deletion mutant Δ*mmpS4/S5* produced much less cell-associated and secreted siderophores compared to wt *Mtb*, but was still capable of synthesizing siderophores in contrast to the Δ*mbtD*::*hyg* mutant (Fig. 3). It should be noted that MBT was detected in the culture supernatants of *Mtb* in addition to cMBT. This is most likely caused by partitioning of cell surface-associated MBT with the medium in the presence of detergents. Taken together, these results suggest that MmpS4 and MmpS5 are part of siderophore export system of *Mtb*.

### MmpS4 and MmpS5 do not appear to be involved in lipid biosynthesis and lipid transport in *M. tuberculosis*

MmpS4 in *M. smegmatis* was previously shown to be involved in biosynthesis and export of glycopeptidolipids (GPLs) [20] which *Mtb* does not synthesize. To examine whether the deletion of *mmpS4* and *mmpS5* caused an altered lipid profile, we performed a complete lipid analysis by TLC (Figs. S9, S10). All major lipids of *Mtb* were identified in wt *Mtb* and the Δ*mmpS4/S5* mutant (Fig. S9, S10) indicating that MmpS4 and MmpS5 are not involved in lipid biosynthesis. However, a lipid which was shown to be produced by *Mtb* under iron limiting conditions [21] was not identified in the Δ*mmpS4/S5* mutant (Figs. S10A–C). Bacon et al. [20] showed by ^1^H-NMR that this lipid consists of a long alkyl chain with a cis double bond and an ester unit. It is unclear whether the absence of this lipid is a direct consequence of the lack of MmpS4/S5, or might be caused indirectly by the slow growth of the double mutant under iron-limiting conditions.

### MmpS4 and MmpS5 are membrane-associated proteins

**Figure 4:**
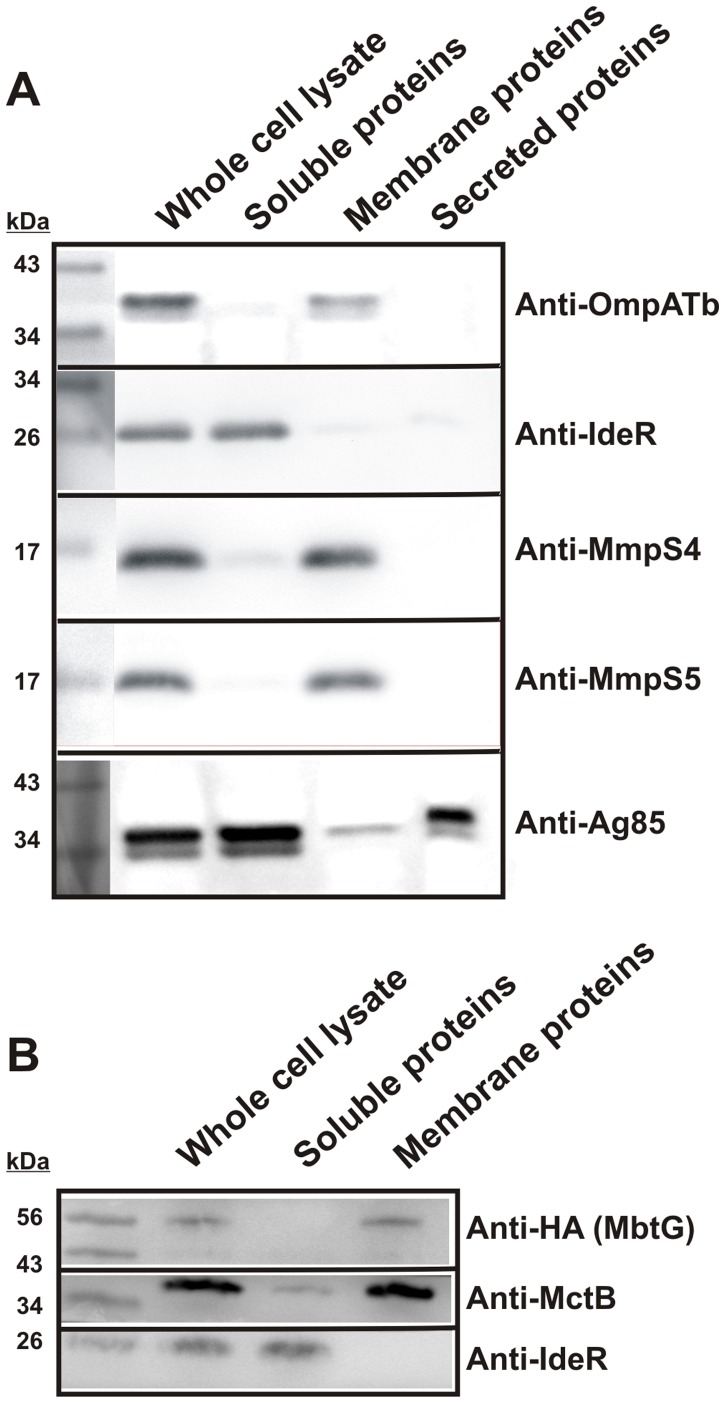
MmpS4, MmpS5 and MbtG are membrane-associated proteins. Proteins of subcellular fractions of wt *Mtb* (ML878) were extracted with 3% SDS and analyzed by SDS-polyacrylamide (10%) gel electrophoresis and Western blot using protein specific antibodies. **A.** Subcellular localization of MmpS4 and MmpS5. OmpATb, IdeR and Ag85 were used as controls for membrane, water-soluble cytoplasmic or periplasmic proteins and secreted proteins, respectively. MmpS4 and MmpS5 were detected using rabbit polyclonal antibodies. **B.** Subcellular localization of MbtG. MctB and IdeR were used as controls for membrane and water-soluble cytoplasmic or periplasmic proteins, respectively. MbtG was expressed with a C-terminal fusion of the Human influenza hemagglutinin (HA) tag which was detected using an HA-specific antibody.

Our data suggests that MmpS4 and MmpS5 are involved in siderophore export, but it is unclear how MmpS4 and MmpS5 contribute to MBT transport. Proteomic analysis of subcellular fractions of *Mtb* yielded contradictory results regarding the localization of MmpS4 and MmpS5 [22], [23]. In order to determine the subcellular localization of MmpS4 and MmpS5, the culture filtrate containing secreted proteins was prepared. Membrane and cytoplasmic fractions were obtained by ultracentrifugation of cell lysates of *Mtb*. Both MmpS4 and MmpS5 were present in the membrane, but not in the cytoplasmic or secreted fractions (Fig. 4A). All fractions were well separated as indicated by the control proteins, the membrane-associated OmpATb (Rv0899), the cytoplasmic regulator IdeR and the secreted Ag85 protein (Fig. 4A). Thus, MmpS4 and MmpS5 are the first examples of membrane-associated proteins that are required for export of siderophores in *Mtb*.

### Membrane-association of MbtG indicates a link between siderophore synthesis and export

The strongly reduced MBT/cMBT level is a striking phenotype considering the intact biosynthesis capacity of the *Mtb* Δ*mmpS4/mmpS5* mutant. Based on the previous observation that MmpS4 connects glycopeptidolipid biosynthesis enzymes with the MmpL4 transporter in *M. smegmatis* [20] we hypothesized that MmpS4 might provide a link between MBT/cMBT biosynthesis and export in *Mtb*. However, *in vivo* crosslinking experiments with formaldehyde in the avirulent *Mtb* strain mc^2^6230 (Table S1) expressing a chromosomal copy of a gene encoding hexahistidine- and HA-tagged MbtG did not show direct binding of MbtG to MmpS4. Next, we examined the subcellular localization of MbtG, the lysine monooxygenase which activates MBT/cMBT by hydroxylating dideoxymycobactins as the predicted last step in MBT biosynthesis [24]. In order to catalyze this reaction MbtG has to be in the cytoplasm because it requires access to the cytoplasmic co-factors NADPH and FAD+. Subcellular fractionation experiments in wt *Mtb* mc^2^6230 revealed that MbtG is membrane-associated although no transmembrane helices and no signal peptide are apparent (Fig. 4B). This result indicates that MbtG might fractionate with membranes due to interactions with another protein and provides the first hint how MBT/cMBT biosynthesis and export might be coupled in *Mtb*.

### MmpS4 and MmpS5 interact with MmpL proteins

**Figure 5:**
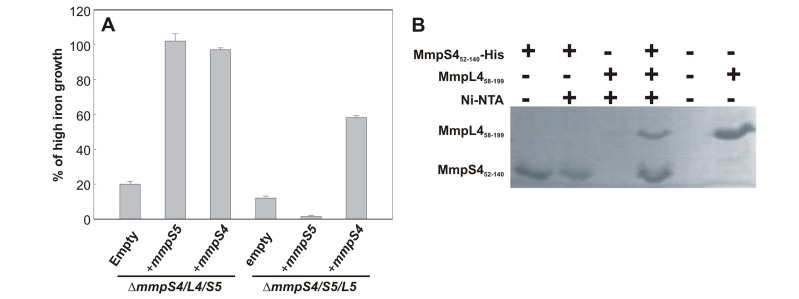
MmpS proteins interact with MmpL proteins. **A.** Genetic interactions between MmpS4 and MmpS5 proteins and their cognate MmpL proteins. Percent of growth in iron-restricted medium (7H9 medium containing 50 µM 2,2′-dipyridyl) of triple mutants Δ*mmpS4/L4/S5* and Δ*mmpS4/S5/L5* strains and those strains complemented with *mmpS4* or *mmpS5* compared to growth in iron-rich media. **B.** Interaction of the C-terminal soluble domain of MmpS4 (residues 52–140) with the L1 loop of MmpL4 (residues 58–199) by an *in vitro* pull down assay.

The *mmpS* genes are located in operons with *mmpL* genes [25]. In order to genetically determine if MmpS4 and MmpS5 interact with their cognate MmpL proteins, the triple mutants Δ*mmpS4/L4/S5* and Δ*mmpS4/S5/L5* were constructed from the Δ*mmpS5* and Δ*mmpS4* strains, respectively, by deleting the respective *mmpSL* operon (Fig. S11). Similar to the double deletion mutant Δ*mmpS4/S5*, these triple mutants failed to grow in iron-deplete media (Fig. 5A). To this end, each triple mutant was complemented with either an empty integrative vector or integrative vectors containing either *mmpS4* or *mmpS5*. The *mmpL5* containing strain (Δ*mmpS4/L4/S5)* complemented with either *mmpS4* or *mmpS5* grew in low iron medium (Middlebrook 7H9 supplemented with 50 µM DIP) (Fig. 5A). However, the *mmpL4* containing strain (Δ*mmpS4/S5/L5*) was only complemented with *mmpS4* but not with *mmpS5*. These results indicate that MmpL4 only interacts with its cognate MmpS4 protein, while MmpL5 is capable of interacting with MmpS4 and MmpS5 to mediate siderophore export by *Mtb*.

To confirm and further define the interaction between MmpS4 and MmpL4, an *in vitro* pull-down assay was employed. According to the topology predictions MmpS4 possesses an N-terminal transmembrane (TM) helix and a C-terminal soluble domain, while MmpL4 contains eleven TM helices and two long loops—L1 between TM1 and TM2, and L2 between TM6 and TM7 (Fig. S12). We tested the interaction between the purified soluble domains of MmpS4 (residues 52–140) and the predicted loops L1 (58–199) and L2 (416–763) of MmpL4. The soluble domain of MmpS4 formed a complex with loop L1 (Fig. 5B), but not with loop L2 (data not shown) of MmpL4. The *in vitro* interaction of the soluble domain of MmpS4 with loop L1 of MmpL4 also shows that both peptides form independently folding domains.

### Structure of MmpS4

**Figure 6:**
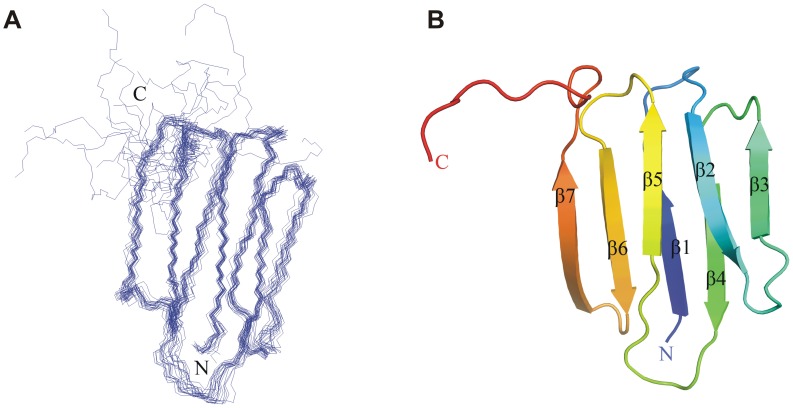
Structure of the C-terminal soluble domain of MmpS4 (residues 52–140). **A.** NMR structure of MmpS4_52–140_ showing the backbone superposition of the final 20 conformers. The coordinates for the structures have been deposited in the Protein Data Bank (PDB accession code 2LW3). **B.** Cartoon depiction of a representative structure.

The *mmpS4* gene encoding an N-terminally truncated MmpS4 protein lacking the predicted transmembrane helix was expressed in *E. coli* and the water-soluble domain of MmpS4 (residues 52–140) was purified by chromatography. The structure of MmpS4_52–140_ was solved by NMR using 762 nuclear Overhauser effect (NOE) and 127 paramagnetic relaxation enhancement (PRE) distance restraints, and 122 dihedral angle restraints (Table S4). The 20 lowest energy structures were selected out of 200 accepted structures. The statistics about the quality and precision of these structures is summarized in Table S4. The backbone superimposition of the final 20 conformers and the representative structure are presented in Fig. 6A. The MmpS4 structure shows seven consecutive β-strands and an unstructured C-terminus (residues 131–140) (Fig. 6B) which might be due to the lack of resonance assignment in this region. The seven β-strands are arranged in two layers, with β4-β1-β6-β7 in one layer and β3-β2-β5 in the other layer.

### MmpS4 and MmpS5 are required for virulence of *M. tuberculosis*

**Figure 7:**
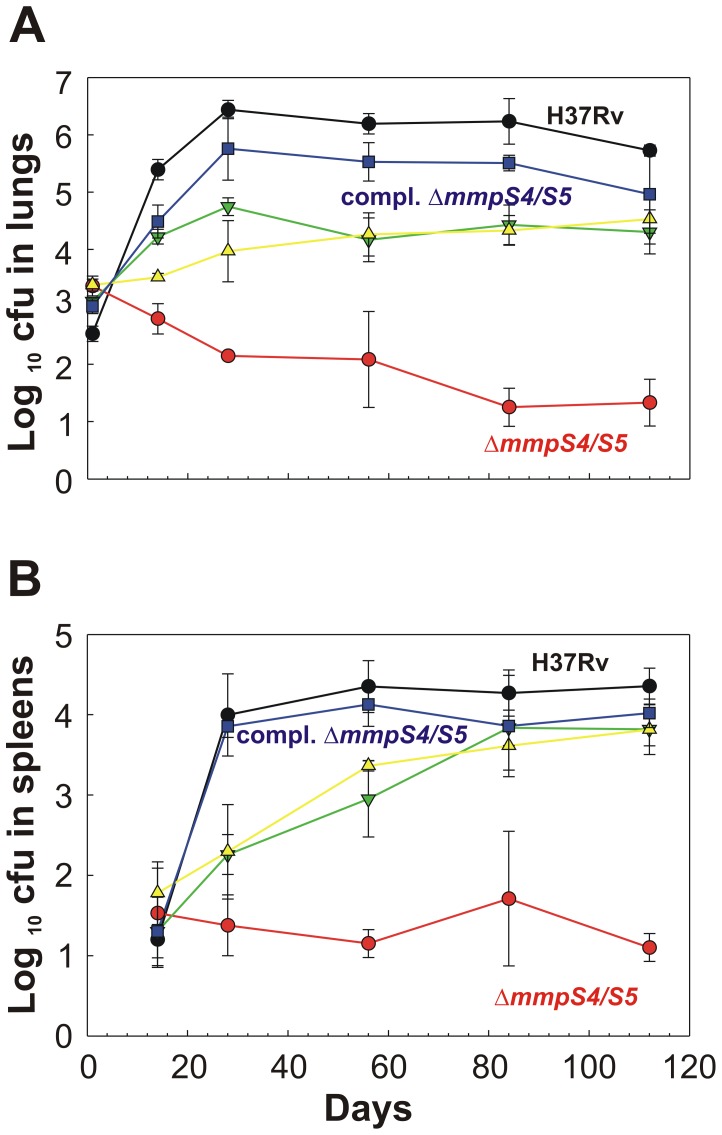
MmpS4 and MmpS5 are required for virulence of *M. tuberculosis* in mice. Colony forming unit (CFU) counts in lungs (**A**) and spleens (**B**) of mice infected with either the wt *Mtb* H37Rv parent strain ML617 (brown circles), the Δ*mmpS4/S5* double deletion mutant ML618 (orange circles), the Δ*mmpS4/S5* mutant singly complemented with *mmpS5* ML619 (olive upside down triangles), the Δ*mmpS4/S5* mutant singly complemented with *mmpS4* ML620 (green triangles), or the Δ*mmpS4/S5* mutant fully complemented with both *mmpS4* and *mmpS5* ML624 (aqua squares). Each data point represents the average of CFUs from the organs of four mice with standard deviations shown.

**Figure 8:**
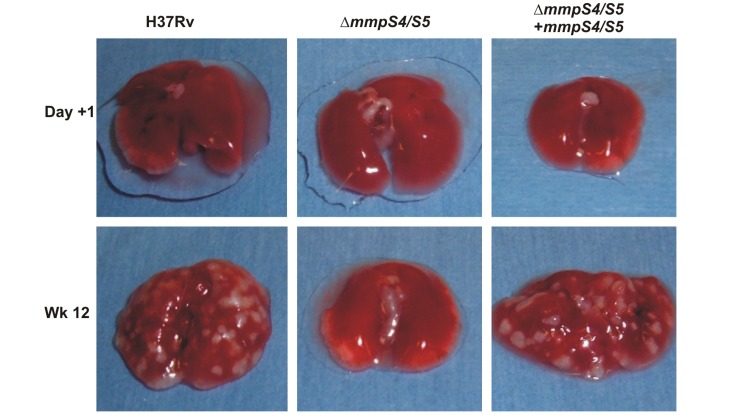
Gross pathology of mouse lungs infected with *M. tuberculosis*. Gross pathology of whole lungs of BALB/c mice infected with wt *Mtb* H37Rv (ML617), Δ*mmpS4/S5* (ML618), and Δ*mmpS4/S5* complemented with both *mmpS4* and *mmpS5* (ML624).

To assess the role of MmpS4 and MmpS5 for virulence of *Mtb*, BALB/c mice were infected with low dose aerosols containing the *Mtb* H37Rv parent strain ML617, the Δ*mmpS4/S5* mutant (ML618), and the double deletion mutant complemented with *mmpS5* (ML619), *mmpS4* (ML620), and *mmpS4/S5* (ML624). The growth kinetics of the parent *Mtb* H37Rv strain in lungs showed the expected logarithmic increase during the acute phase followed by a plateau during the chronic phase of infection. Similar growth kinetics in spleens demonstrated that this strain is competent for dissemination. Loss of the single *mmpS4* and *mmpS5* genes also compromised the ability of *Mtb* to survive in the lungs as the number of viable bacteria decreased by 100-fold from the initial burden compared to wt *Mtb*. However, loss of these genes alone did not alter the ability of *Mtb* to disseminate to and proliferate in the spleen. The Δ*mmpS4/S5* mutant failed to proliferate in lungs and spleen as reflected by a 24,000- and 1,800- fold, respectively, decreased bacterial burden compared to wt *Mtb* after 16 weeks of infection (Fig. 7). Loss of these genes resembles the “severe growth *in vivo*” (*sgiv)* phenotype [26] and, to our knowledge, is the strongest *in vivo* phenotype observed so far for genes involved in iron utilization by *Mtb*. The single *mmpS4* or *mmpS5* genes partially complemented the virulence defect of the double mutant. Full complementation of the double mutant by both genes confirmed that the *mmpS4* and *mmpS5* genes are essential for virulence of *Mtb* (Fig. 7). Gross mouse lung examination and histological assessment in mice infected with the Δ*mmpS4/S5* double deletion strain showed almost no signs of infection (Figs. 8, S13–15). However, lungs of mice infected with either *Mtb* H37Rv wt or the fully complemented Δ*mmps4/S5* strain exhibited extensive lesions (Figs. 8, S13) and displayed significant lymphocytic infiltrates (Fig. S14). Lungs of mice infected with the *mmpS4* or *mmpS5* singly complemented strains showed lesions and lymphocytic infiltrates, but to a much lesser degree than lungs of mice infected with wt or the fully complemented strain.

**Figure 9:**
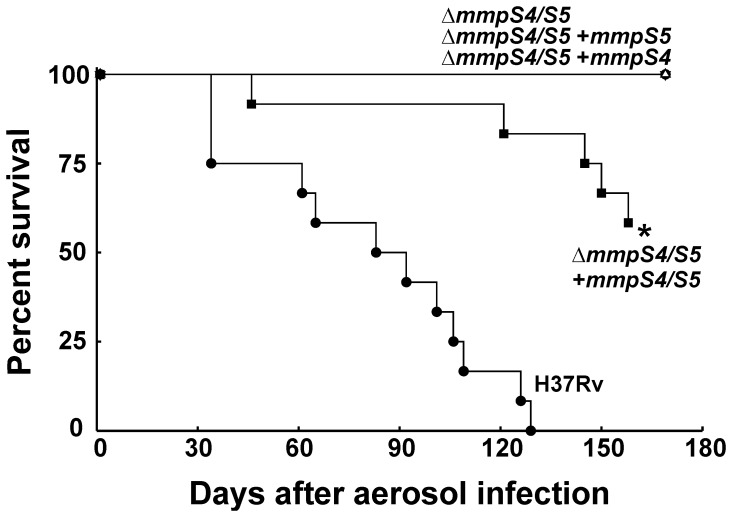
Effect of *mmpS4* and *mmpS5* on the survival of mice infected with *M. tuberculosis*. Survival of mice infected with wt *Mtb* H37Rv (ML617), Δ*mmpS4/S5* (ML618), Δ*mmpS4/S5* singly complemented with *mmpS5* (ML619), Δ*mmpS4/S5* singly complemented with *mmpS4* (ML620), or Δ*mmpS4/S5* fully complemented with *mmpS4* and *mmpS5* (ML624). Thirteen mice were infected with each strain. Mice were euthanized at day 169.

In survival experiments loss of *mmpS4* and *mmpS5* severely attenuated virulence of *Mtb* as none of the mice infected with the Δ*mmpS4/S5* double deletion mutant died within 180 days (Fig. 9). Similarly, loss of either *mmpS4* or *mmpS5* alone resulted in attenuation of virulence. The difference in mean survival time between the groups of mice infected with wt and the fully complemented strain was longer than expected and could partly be explained by a lower bacterial burden of the fully complemented strain in the lungs. In conclusion, the infection experiments revealed that *mmpS4* and *mmpS5* are essential for virulence of *Mtb* in mice.

## Discussion

### MmpS4 and MmpS5 in complex with their cognate MmpL proteins constitute siderophore export systems in *M. tuberculosis*

In this study, we showed that the lack of MmpS4 and MmpS5 strongly reduced siderophore secretion and caused a growth defect of *Mtb* under low iron conditions. Pull-down experiments demonstrated that the MmpS4 protein forms a complex with the inner membrane transporter MmpL4 *in vitro*. This observation was corroborated by genetic complementation experiments demonstrating that MmpS4 and MmpS5 interact with their respective MmpL proteins to restore growth of *Mtb* under iron-limiting conditions. Considering that siderophore uptake is not altered in *Mtb* lacking *mmpS4/S5* and that the MmpL proteins are inner membrane transporter proteins, it is concluded that the respective MmpS/MmpL complexes translocate siderophores across the inner membrane into the periplasmic space. Such a transport process is defined as export [27].

Proteins which enable siderophore export in *Mtb* have been unknown so far [11], largely because *Mtb* does not have any proteins resembling known siderophore export systems such as EntS of *Escherichia coli* [13] or PvdE of *Pseudomonas aeruginosa* [28]. Previously, MmpS5 and MmpL5 have been implicated in drug efflux due to weak similarity with RND efflux pumps of *E. coli* [29]. MmpL3, MmpL7 and MmpL8 were shown to export lipids such as trehalose monomycolate [30], [31], [32], phthiocerol dimycocerosate [33], [34], and sulfolipid 1 [35] leading to the hypothesis that the MmpL proteins are lipid transporters. Since carboxymycobactins and in particular mycobactins are quite hydrophobic molecules and have similar chemical properties as lipids, this finding is rather an expansion than a deviation from the rule. Taken together, we conclude that MmpS4/MmpL4 and MmpS5/L5 constitute novel bacterial siderophore export systems.

### The reduced siderophore levels in the *mmpS4/S5* double mutant suggest a link between siderophore biosynthesis and export in *M. tuberculosis*

The lack of the MmpS4/5 proteins also reduced the amount of detectable carboxy/mycobactins suggesting a role in biosynthesis of these siderophores in *Mtb*. Recently, a biosynthetic pathway comprising all enzymatic activities required for MBT/cMBT biosynthesis has been proposed based on the substrate specificities of enzymes encoded by the *mbt* operons [16]. Modifying enzymes to generate nonmethylated or α-methylated MBT derivatives have not been identified yet, but they are not expected to alter the total MBT amount. Thus, it is concluded that the strongly reduced siderophore levels in the *Mtb* Δ*mmpS4/S5* mutant likely result from an indirect effect of these proteins on biosynthesis. Indeed, such a mechanism has been proposed for the MmpS4 protein which is required for efficient synthesis and export of surface-exposed glycopeptidolipids (GPL) in *M. smegmatis* [20]. Co-localization of MmpS4 with FadD23 and MbtH indicated that the GPL biosynthesis enzymes form a multi-protein complex with the membrane proteins MmpS4 and MmpL4a/b in *M. smegmatis*. Since lack of MmpS4 resulted in enzyme diffusion in the cytoplasm, biosynthesis of GPLs was much less efficient in *M. smegmatis* [20]. This phenotype was complemented by the *Mtb mmpS4* gene indicating that *Mtb* MmpS4 also enables formation of a biosynthetic multi-enzyme complex at the inner membrane of *M. smegmatis*. In this study we show, that MmpS4 is involved in siderophore export in *Mtb*. The fact that the siderophore biosynthesis enzyme MbtG is located at the inner membrane, as shown in this study, supports the hypothesis that a similar multi-enzyme complex for efficient siderophore synthesis and transport exists in *Mtb*.

In principle, block of transport caused by the *mmpS4/mmpS5* deletions and degradation of siderophores as is observed for the ferric enterobactin esterase IroD, IroE, and Fes of *E. coli* and Salmonella [36] would also explain the low level of MBTs/cMBTs in the *Mtb mmpS4/mmpS5* double mutant. However, there are no enzymes in *Mtb* with similarities to known siderophore esterases. In addition, degradation of imported siderophores to release iron in the cytoplasm is rare and has only been observed for trilactone siderophores such as enterobactin of *E. coli* and *Salmonella* [37]. The high energy cost of MBT/cMBT production [38], [39] also argues in favor of a synthesis tightly regulated by the requirement for iron and the capacity to export newly synthesized siderophores. Coupled synthesis and export would also prevent toxic accumulation of siderophores in *Mtb* as has been observed in other bacteria [40], [41], [42].

### The MmpS4 and MmpS5 proteins do not confer substrate specificity

An interesting question is whether the MmpL4/MmpS4 or the MmpL5/MmpS5 systems are specific for MBTs or cMBTs. The single mutants clearly produce and secrete both siderophores indicating that the MmpS4/MmpS5 proteins are not specific for either substrate. This conclusion is supported by the observation that the *Mtb mmpS4* gene complements the GPL synthesis and transport defect of the *M. smegmatis mmpS4* mutant, although GPLs do not exist in *Mtb* [20]. It is more likely that the transporters themselves, namely MmpL4 and MmpL5, confer specificity for MBTs or cMBTs. This hypothesis is currently under investigation. Interestingly, we observed that both MmpS4 and MmpS5 interact with MmpL5, while MmpS5 cannot restore growth of an *Mtb* triple mutant Δ*mmpS4/S5/L5* expressing only *mmpL4* under iron-limiting conditions. Thus, MmpS4 seems to be more promiscuous in its interactions with MmpL proteins. In this regard, it should be noted that the genetic complementation experiments indicate that the MmpS5/MmpL5 pair is more efficient in restoring wt growth of *Mtb* under low iron conditions. In conclusion, it appears that *Mtb* ensures efficient siderophore export by employing at least two partially redundant transporters.

### NMR experiments revealed a novel structure for accessory proteins in complex transporter systems

The NMR structure of MmpS4_52–140_ revealed no similarity to any protein of known function, but was similar to an uncharacterized protein from *Parabacteroides distasonis* (PDB: 2LGE). The superimposition of the MmpS4 structure with that of this putative calcium-binding protein showed a root-mean-square deviation of 3.6 Å over the Cα atoms of 75 aligned residues (Fig. S15A) with similar secondary and tertiary structures (Fig. S15B). Secondary structure prediction [43] indicated an eighth β-strand including the residues 131–137 of MmpS4. This gave rise to the hypothesis that the C-terminus of MmpS4 might be unordered in its unbound state, but may form a more stable structure with two 4-stranded sheets when bound to MmpL4. Further experiments are required to provide evidence for this hypothesis. Importantly, the structure of MmpS4 shows no similarity to AcrA [44] or other periplasmic adapter proteins from drug efflux systems of Gram-negative bacteria [45] indicating that the mycobacterial MmpS proteins constitute a novel class of accessory proteins in complex transporter systems.

### Model of siderophore–mediated iron uptake by *M. tuberculosis*

**Figure 10:**
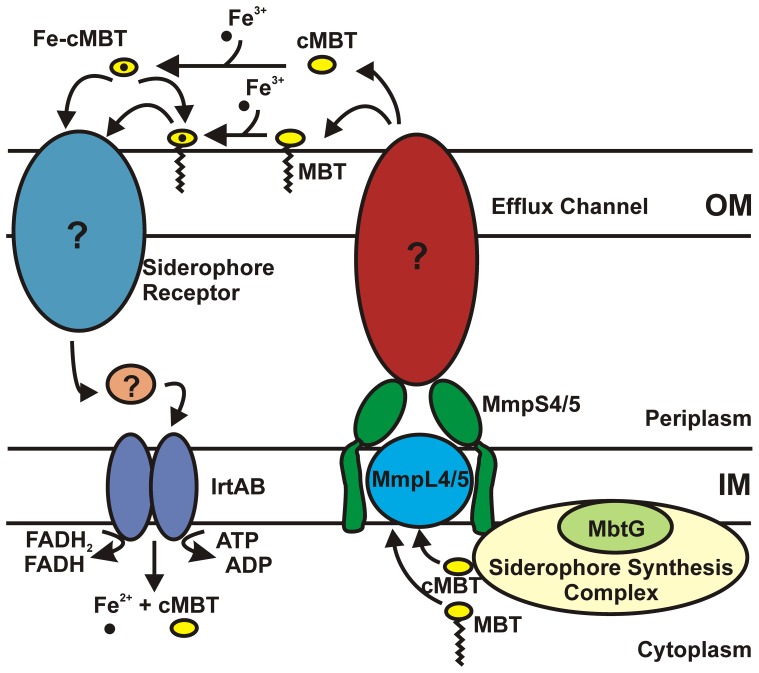
Model of siderophore–mediated iron uptake by *M. tuberculosis*. Siderophores are synthesized by cytoplasmic synthases that function as a complex. Synthesis and transport of siderophores is likely coupled and dependent on the activity of the RND transporters, MmpL4 and MmpL5. Siderophore export requires that the membrane-associated proteins MmpS4 and MmpS5 function together with their cognate MmpL proteins. MmpS4 and MmpS5 are anchored to the inner membrane and may function as periplasmic adaptor proteins. Export of siderophores across the OM would require a yet undiscovered OMP. Once secreted, siderophores bind iron and would require an OMP siderophore receptor for transport across the OM. Once in the periplasmic space IrtAB imports ferric-siderophores across the inner membrane where iron is released from the siderophore and becomes available for the cell.

In this study, we identified a novel siderophore export system of *Mtb* which is composed of the transporters MmpL4 and MmpL5 and their associated MmpS proteins. Previously, it was proposed that the MmpS proteins function as periplasmic adapter proteins [29] which was based on the low sequence similarities between the transporters of tripartite efflux pumps of Gram-negative bacteria [46] with MmpL proteins [47]. The localization of MmpS4 in the periplasm of *M. smegmatis* [20] and our observation that the MmpS4/5 proteins interact with their respective MmpL transporters support their role as accessory transport proteins. In addition, we show that, in contrast to their Gram-negative counterparts, the MmpS4/MmpS5 proteins are not only required for export, but also for biosynthesis of cMBT/MBT. Therefore, we hypothesize that MmpS4 functions as a scaffolding protein to couple synthesis and export of MBT/cMBT in *Mtb* as has been proposed for GPLs in *M. smegmatis* [20]. The surprising result that the MBT/cMBT activating enzyme MbtG is membrane-associated despite the absence of any recognizable membrane anchor domain suggests that MbtG might interact directly or indirectly with membrane proteins such as MmpL4/L5 in *Mtb*. These findings are summarized in the model depicted in Fig. 10. Hitherto unknown are the hypothetical outer membrane proteins required for cMBT/MBT secretion to the extracellular medium and for uptake of cMBT. The role of the Esx-3 system in cMBT/MBT-mediated iron acquisition is also unknown [7].

### Role of mycobactin export in iron homeostasis of *M. tuberculosis*

An interesting observation was that growth of the *Mtb* Δ*mmpS4/S5* mutant under low iron conditions was not fully restored by adding hemoglobin as an iron source (Fig. 1). This result is in contrast to the *Mtb* Δ*mbtD::hyg* mutant which is unable to synthesize mycobactins [18]. However, the Δ*mmpS4/S5* mutant grew like wt *Mtb* with hemoglobin as the sole iron source when MBT/cMBT biosynthesis was additionally eliminated. These results indicate that low level synthesis of siderophores and their intracellular accumulation due to the lack of export inhibits growth of the Δ*mmpS4/S5* mutant, e. g. by chelating iron or other cations from essential proteins of *Mtb*. The mechanism of this peculiar type of growth inhibition is currently under investigation.

### Role of siderophore export in virulence of *M. tuberculosis*

Deletion of both *mmpS4* and *mmpS5* drastically reduced the virulence of *Mtb* in mice. Considering the strong growth defect of the Δ*mmpS4/S5* mutant under low iron conditions *in vitro* and the known requirement of siderophore biosynthesis and utilization for growth of *Mtb in vivo* [5], [6], [7], it is likely that the attenuation of the Δ*mmpS4/S5* mutant is due to its inability to take up sufficient iron in the absence of siderophores. However, it cannot be excluded that other functions of MmpS4 and MmpS5 contribute to the virulence defect of the Δ*mmpS4/S5* mutant. In favor of this conclusion is the observation that expression of *mmpS5* fully restores siderophore export and growth of the *Mtb* Δ*mmpS4/S5* mutant under low iron conditions *in vitro*, but still has a significant virulence defect in mice. This result indicated that other functions of MmpS4, which are not present in MmpS5, may contribute to the virulence defect of the Δ*mmpS4/S5* mutant. Interestingly, the *Mtb mmpL4* mutant showed a 10-fold reduced bacterial burden in the lungs of mice [47]. This is consistent with our finding that the number of bacteria of an *Mtb* strain which lacks only *mmpS4* was between 10- and 100-fold lower in the lungs of mice after the acute phase of infection. Similarly, the slight attenuation of an *Mtb* strain which lacks only *mmpS5* is consistent with the *in vivo* growth defect of an *Mtb mmpS5* mutant in transposon site hybridization (TraSH) studies [48]. The loss of virulence of *Mtb* Δ*mmpS4/S5* mutant in mice is much more pronounced than that observed for the *irtAB* mutant which lacks an ABC transporter required for cMBT uptake [6]. This correlates with the different magnitude of their *in vitro* phenotypes: While the *irtAB* mutant showed only a minor growth defect under low iron conditions [6], loss of *mmpS4* and *mmpS5* completely abolished growth of *Mtb* under those conditions (Figs. 1, S1, S2).

### Conclusions

In this study, we identified that interaction of the membrane proteins MmpS4 and MmpS5 with their cognate MmpL transporters is required for siderophore export in *Mtb* and propose a model for siderophore secretion. These novel siderophore transport systems are essential for virulence of *Mtb* in mice. Considering the almost universal requirement of bacterial pathogens for iron [49] it is tempting to speculate that these systems might be good drug targets. However, more work is required to determine whether these two partially redundant transporters can be poisoned by a single drug.

## Materials and Methods

### Ethics statement

BALB/c mice were obtained from the Charles River Laboratories and were housed and cared for in a pathogen-free biosafety level 3 vivarium facility at Johns Hopkins University. Mice were provided food and water ad libitum as well as appropriate monitoring and clinical care. The protocols used in this study were reviewed and approved by the Johns Hopkins Institutional Animal Care and Use Committee and are described in protocol MO09M101. The Johns Hopkins Animal Care and Use Committee complies with Animal Welfare Act regulations and Public Health Service Policy. Johns Hopkins University also maintains accreditations with the Association for the Assessment and Accreditation of Laboratory Animal Care (AAALAC) International.

### Bacterial plasmids and strains, media, and growth conditions

The strains used in this study are listed in the supplement (Table S1). Media, growth conditions and construction of plasmids are described in detail in the supplement (Text S1).

### Construction of mutants in *Mtb* H37Rv and *Mtb* mc^2^6230

The *mmpS4* (*rv0451c*), *mmpS5* (*rv0677c*), *mmpS4/S5*, *mbtD* (*rv2831c*), and *mmpS4/S5/mbtD* deletion mutants of *Mtb* H37Rv and *Mtb* mc^2^6230 were constructed using a two-step selection strategy as described in SI. Complementation of Δ*mmpS4/S5* double deletion mutant of *Mtb* H37Rv and *Mtb* mc^2^6230 were performed using L5 and Ms6 phage integration systems as described in the supplement (Text S1).

### Drop assay

Low-iron GAS plates were prepared by dissolving 150 mg Bacto Casitone, 2 g K_2_HPO_4_, 1 g citric acid, 0.5 g L-alanine, 0.6 g MgCl_2_•6H_2_0, 0.3 g K_2_SO_4_, 1 g NH_4_Cl, and 8.3 ml 60% glycerol in 450 ml high grade Millipore water (Barnstead Nanopure Diamond; 18.2 MΩ-cm), the pH was adjusted to 6.6 with NaOH and 5 g Agar Noble (BD Biosciencese) was added. The volume was brought up to 500 ml in an acid washed glass bottle (6 M HCl), autoclaved, supplemented with pantothenate, hygromycin, and split into acid washed bottles to which 5 µM human hemoglobin was added when required. Pre-cultures were grown in 7H9 Middlebrook medium supplemented with 10% OADC, 0.2% casamino acids, 24 µg/ml pantothenate, 50 µg/ml hygromycin, 0.02% tyloxapol (7H9 MR) and 20 µM hemin. Once in mid-logarithmic phase (OD_600_ = 0.5–2.0) cells were filtered through a filter with 5 µm pores and washed once in low-iron GAS medium. Cells were diluted to an OD_600_ = 0.01 and 10-fold serial dilutions were prepared in low-iron GAS medium. 3 µl of each dilution was deposited on low-iron GAS plates or low-iron + hemoglobin plates using a multi-channel pipette. Plates were incubated for nine weeks at 37°C.

### IdeR reporter assay

Strains were grown in 7H9 MR medium. Cultures were inoculated in biological triplicate, grown at 37°C and split at mid-logarithmic phase (OD_600_ = 0.2–0.3). Purified Fe-cMBT-BCG (1 µg/ml) was added to one set of triplicates, while other triplicates were left untreated. Optical densities were determined in 1 cm path length cuvettes by diluting cells to OD_600_ = 0.1–1 in the above media. Readings were taken every day until stationary phase was reached. Fluorescence intensities reported in Fig. 2A were two days after the addition of Fe-cMBT.

Green fluorescent protein (GFP) fluorescence intensities were determined using a Biotek Synergy HT plate reader with a 485 nm excitation and a 528−/+20 nm emission filter. Fluorescence intensities were normalized to the optical density of the same samples according to the following equation:
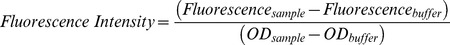



### Uptake of carboxymycobactin by *Mtb*

Fe-cMBT-BCG (93 µg) was deferrated as previously described by incubation in the presence of 50 mM EDTA pH = 4.0 at 37°C for 18 hours [8]. Precipitated EDTA was pelleted by centrifugation, supernatant was extracted twice with chloroform, washed twice with water and evaporated to dryness. Deferrated residue was suspended in a 1∶1 mixture of EtOH and 50 mM KH_2_PO_4_ buffer pH = 7.0. ^55^Iron- (396 µCi) was added to the mixture and incubated for 1 hour at room temperature (at which point, the solution developed a brown hue). One ml of water was added to the mixture and extracted twice with 2 volumes of chloroform. The chloroform extract was washed twice with water and evaporate to dryness. The material was resuspended in warm ethanol. This preparation yielded 16.2 µM ^55^Fe-cMBT-BCG with the radioactive concentration of 47.5 µCi/ml.

The strains Δ*mbtD*::*hyg* and Δ*mmpS4/S5/*Δ*mbtD*::*hyg* were grown in 7H9 MR medium, and 20 µM hemin to OD_600_ = 1.0. Cells were washed on ice with a low iron media consisting of 500 µM MgCl_2_•6H_2_0, 7 µM CaCl_2_•2H_2_O, 1 µM NaMoO_4_•2H_2_O, 2 µM CoCl_2_•6H_2_O, 6 µM MnCl_2_•4H_2_O, 7 µM ZnSO_4_•7H_2_O, 1 µM CuSO_4_•5H_2_O, 15 mM (NH_4_)_2_SO_4_, 12 mM KH_2_PO_4_ pH = 6.8, 1% (w/v) glucose, which was supplemented with 10% OADC, and 0.2% casamino acids. Cells were resuspended in the same media to an OD_600_ of approximately 3.0 on ice. For uptake experiments, 2 ml of cell suspensions were equilibrated at 37°C for 15 min and shaken at approximately 400 rpm. ^55^Fe-labeled cMBT was added to the cells at a final concentration of 0.25 µM cMBT, 0.45 µCi ^55^Fe. 200 µl samples were removed at 1, 2, 4, 8, and 16 minutes and added to 400 µl of a killing buffer consisting of 100 mM LiCl, 50 mM EDTA in 4% formaldehyde in Spin-X filter microcentrifuge tubes. Cells were immediately centrifuged and washed twice in killing buffer. The radioactivity of the cells was quantified using liquid scintillation counting (Beckman Coulter LS6500). ^55^Fe counts were converted to total iron by the use of a standard curve and normalized to dry weight of cells by determining the dry mass of 4 ml of the washed cell suspensions. All experiments were done in triplicate.

### Radiolabelling of *Mtb* siderophores

Radiolabelling of siderophores was performed in a similar manner as previously described with modifications [5], [50]. Iron free self-made 7H9 media supplemented with 0.2% glucose and 0.01% Tyloxapol was deferrated using Chelex-100 to remove any trace contaminants of iron. Pre-cultures were grown under iron rich conditions to OD_600_ of 1–2. To deplete intracellular iron stores, iron free media was inoculated with pre-culture to an OD_600_ = 0.05, and allowed to grow to OD_600_ of 1–2. Only ML618 (Δ*mmpS4/S5*) and ML1424 (Δ*mbtD*::*hyg*) were not grown in iron free media because these strains do not grow under low iron conditions; however, IdeR derepression occurs even under iron-replete conditions in these strains (this study). Five ml of cell cultures were adjusted to OD_600_ = 0.2 using iron free media and incubated with 1 µCi/ml [7-^14^C]-salicylic acid (21.3 µM final concentration) for 11 days while shaking at 37°C. Cultures were centrifuged at 4,000× g for 7 min and supernatants and cell pellets were collected. Ferric chloride (20 mg/ml FeCl_3_•6H_2_O in ethanol) was added to the supernatants at a final concentration of 0.6 mM and allowed to incubate at room temperature for one hour. The supernatants were extracted twice with 5 ml CHCl_3_ and the organic fraction was retained. The cell pellets were resuspended in 2.5 ml of ethanol and incubated with shaking for 12 hours at 37°C. After centrifuging for 7 min at 4,000× g, the ethanol supernatant was retained and 2.5 ml of water and FeCl_3_ (to 2.2 mM) was added. This mixture was allowed to incubate at room temperature for one hour, after which it was extracted twice with 5 ml CHCl_3_ and the organic fraction retained. The cell and supernatant extracts were then evaporated using a Vacuufuge (Eppendorf) and resuspended in 500 µl CHCl_3_. After having normalized based on CPMs, extracts were then subjected to TLC on 10 cm×10 cm, 250-µm-thick silica gel 60 (Sigma) developed in ethanol/hexanes/water/ethyl acetate/acetic acid, 5∶25∶2.5∶35.5 [51]. Plates were allowed to dry and then exposed to a phosphoimager screen for 60 hours and analyzed with a Storm Phosphoimager (Molecular Dynamics). ^55^Fe-loaded MBT and cMBT, as well as radiolabelled salicylic acid substrate, were also subjected to TLC alongside the extracts. *R*_f_ values for MBT (0.42) and cMBT (0.16) were the same as those previously reported and the salicylic acid, MBT, and cMBT loading controls ran the same as their extracted radiolabelled counterparts.

### Virulence studies in mice

Prior to virulence studies, all strains were demonstrated to have PDIMs and positive neutral red assessments. Mid-log phase cultures of wt *Mtb* (ML617), the *mmpS4/S5* double deletion mutant (ML618), the *mmpS5* singly complemented mutant (ML619), the *mmps4* singly complemented mutant (ML620), and the doubly *mmpS4/S5* complemented mutant (ML624) were diluted to OD_600_ ∼0.1 to implant ∼1,000 bacilli in the lungs of mice using a Middlebrook inhalation exposure system (Glas-Col). Twenty four 4- to 5-week old female BALB/c mice (Charles River) were infected with ML617, ML618, ML619, ML620, or ML624. Four mice from each group were weighed and sacrificed at days 1, 14, 28, 56, 84, and 112 post-infection to determine the number of bacilli in the lung and spleen. Mouse organs were aseptically removed, homogenized, and serially diluted. Appropriate dilutions were plated onto Middlebrook 7H11 agar plates to determine the colony forming units. For histological analysis, representative tissue samples from each group at days 1, 28, 56, 112 post-infection were fixed in 10% formaldehyde, embedded in paraffin, sectioned, and stained with hematoxylin and eosin using standard procedures. Thirteen 4- to 5-week old female BALB/c mice were infected with 7,500–10,000 bacilli using the five strains mentioned above using the same method already described. Time to death was followed and survival proportions of mice infected with high dose aerosol were calculated. The experiment was stopped after 180 days post-infection.

Detailed protocols for other experiments are provided in the supplement (Text S1).

## Supporting Information

Figure S1 **Growth of** ***Mtb*** **in low iron media.** Growth of wt *Mtb*, Δ*mmpS4*, Δ*mmpS5*, Δ*mmpS4/S5*, fully complemented Δ*mmpS4/S5* (+*mmpS4/S5*) and Δ*mbtD*::*hyg* in low iron HdB media supplemented with 10% OADC, 0.2% casamino acids, 24 µg/ml pantothenate, and 0.02% tyloxapol without (**A**) and with (**B**) 20 µM hemin as an iron source.
(TIF)

Figure S2 **Growth of** ***Mtb*** **in 7H9 media supplemented with 2,2′-dipyridyl.** **A.** Growth of wt *Mtb*, Δ*mmpS4*, Δ*mmpS5*, Δ*mmpS4/L4*, Δ*mmpS5/L5*, Δ*mmpS4/S5*, and Δ*mbtD*:: *loxP* strains in iron-replete 7H9 media supplemented with 10% OADC, 0.2% casamino acid, 24 µg/ml pantothenate, and 0.01% tyloxapol. Strains were started out at an OD_600_ = 0.05 and optical densities were measured at regular intervals for 15 days. Strains were grown in triplicate and standard deviations are shown. **B.** Growth of wt *Mtb*, Δ*mmpS4*, Δ*mmpS5*, Δ*mmpS4/L4*, Δ*mmpS5/L5*, Δ*mmpS4/S5*, and Δ*mbtD*::*loxP* strains in iron-deplete 7H9 media supplemented with 10% OADC, 0.2% casamino acids, 24 µg/ml pantothenate, 0.01% tyloxapol, and 0.1 mM 2,2′-dipyridyl (DIP) as an iron chelator. Strains were grown in triplicate and standard deviations are shown. **C.** Either *mmpS4* or *mmpS5* rescues the low iron delayed growth phenotype of Δ*mmpS4/S5*. Low iron growth in the presence of 0.1 mM DIP is reported as the percentage of growth in iron-replete media. Wild-type *Mtb* (ML617), Δ*mmpS4/S5* (ML618), *mmpS5* singly complemented Δ*mmpS4/S5* (ML619), *mmpS4* singly complemented Δ*mmpS4/S5* (ML620), and Δ*mmpS4/S5* fully complemented with *mmpS4* and *mmpS5* (ML624), were grown in both iron-replete 7H9 media and iron-deplete 7H9 supplemented with DIP.
(TIF)

Figure S3 **Southern blot analysis of deletion mutants in virulent** ***Mtb*** **.** Chromosomal DNA of *Mtb* strains were digested with *Aat*II, *Apa*I, or *Nru*I, for analysis of *mmpS4*, *mmpS5* and *mbtD* genomic regions, respectively. Digested chromosomal DNA was analyzed by Southern blotting using probes generated by PCR from genomic DNA. In frame deletions of *mmpS4* and *mmpS5* were constructed through homologous recombination and subsequent excision by Cre recombinase of the *hyg* marker. In the *mmpS4* and *mmpS5* deletion mutants, 373 and 363 bp, respectively, were replaced by *loxP* sites such that no stop codons were introduced into the reading frames. ML617 is the *Mtb* H37Rv parent wt strain. ML472 is the Δ*mmpS4* single deletion strain. ML405 is the Δ*mmpS5* single deletion strain. ML618 is the Δ*mmpS4/S5* double deletion strain. ML1424 is the Δ*mbtD*::*hyg* deletion strain.
(TIF)

Figure S4 **Expression of** ***mmpS4*** **and** ***mmpS5*** **in** ***M. tuberculosis*** **.** **A.** Expression of *mmpS4* and *mmpS5* in virulent *Mtb* strains. Proteins were extracted with 1% SDS from wt *Mtb* (ML617), the Δ*mmpS4* single deletion mutant (ML472), the Δ*mmpS5* single deletion mutant (ML405), the Δ*mmpS4/S5* double deletion mutant (ML618), the Δ*mmpS4/S5* mutant singly complemented with *mmpS5* (ML619), the Δ*mmpS4/S5* mutant singly complemented with *mmpS4* (ML620), and the Δ*mmpS4/S5* mutant fully complemented with *mmpS4* and *mmpS5* (ML624). *MmpS4* and *mmpS5* were complemented using the expression vectors, pML1545 and pML1560, respectively. Proteins were detected in a Western blot by using rabbit polyclonal antibodies raised against MmpS4 and MmpS5. As a loading control, RNA polymerase (RNAP) was detected using anti-RNAP β subunit (Neoclone). **B.** Expression of MmpS4 and MmpS5 in avirulent *Mtb* strains. Strains include wt *Mtb* (ML878), the Δ*mmpS4* single deletion mutant (ML475), the Δ*mmpS5* single deletion mutant (ML406), the Δ*mmpS4/S5* double deletion mutant (ML1401), the Δ*mmpS4/S5* mutant singly complemented with *mmpS5* (ML886), the Δ*mmpS4/S5* mutant singly complemented with *mmpS4* (ML887), and the Δ*mmpS4/S5* mutant fully complemented with *mmpS4* and *mmpS5* (ML889). The same methods were used as in fig. S4A.
(TIF)

Figure S5 **Growth of** ***Mtb*** **in 7H9 media containing desferrioxamine.** Growth of wt *Mtb* (A), Δ*mbtD::loxP* (B) and Δ*mmpS4/S5* (C) in 7H9 media supplemented with 10% OADC, 0.2% casamino acids and 24 µg/ml pantothenate containing 0 (black), 100 (red), or 200 µM (green) concentrations of the ferric specific chelator desferrioxamine (DFO). Strains were started out at an OD_600_ = 0.05 and optical densities were measured at regular intervals for 17 days. Strains were grown in triplicate and standard deviations are shown.
(TIF)

Figure S6 **Growth of Δ** ***mmpS4/S5*** **in 7H9 media containing 2,2′-dipyridyl.** Growth of Δ*mmpS4/S5* in 7H9 media supplemented with 10% OADC, 0.2% casamino acids and 24 µg/ml pantothenate containing 0 (black), 10 (red), 20 (green), 40 (yellow), or 80 (blue) µM concentrations of 2,2′-dipyridyl. Strains were started out at an OD_600_ = 0.05 and optical densities were measured at regular intervals for 9 days. Strains were grown in triplicate and standard deviations are shown.
(TIF)

Figure S7 **Validation of a cytoplasmic iron reporter in** ***Mtb*** **mc^2^6230.** *Mtb* mc^2^6230 was grown in low iron HdB supplemented with 10% OADC, 0.2% casamino acids, 24 µg/ml pantothenate, 0.02% tyloxapol, and indicated ferric citrate concentrations. Fluorescence readings were taken at indicated time points as described in materials and methods.
(TIF)

Figure S8 ***M. tuberculosis mbtD*** **mutants used in this work.** **A.** Genomic region encompassing *mbtD* in *M. tuberculosis* wt, Δ*mbtD*::*hyg* and Δ*mbtD::loxP*. **B.** Low iron growth phenotypes of wt, Δ*mbtD*::*hyg*, and Δ*mbtD::loxP*. Strains were grown in low iron HdB supplemented with 10% OADC, 0.2% casamino acids, 24 µg/ml pantothenate and 0.02% tyloxapol.
(TIF)

Figure S9 **Lipid analysis of** ***mmpS4*** **and** ***mmpS5*** **mutants of** ***M. tuberculosis*** **by thin layer chromatography.** Extracts of the indicated *M. tuberculosis* strains containing polar (**A**) and apolar (**B**, **C**, **D**) lipids were analyzed by thin layer chromatography (TLC). The amount of loaded lipids in A corresponds to 0.2 mg of delipidated cells, while the amounts loaded in B, C, D correspond to 0.4 mg. The TLC plates A and B were resolved using solvent system a. The TLC plate C was resolved using solvent system b and the plate D was resolved by solvent system c. Plate A shows phospholipids visualized by the Dittmer-Lester reagent. Anthrone was used to visualize sugar-containing lipids on plates B and D. Plate C shows trehalose mycolates visualized by copper sulfate in phosphoric acid. As a control, the first lane shows 4 mg of trehalose-di-mycolates (Sigma). TDM, trehalose dimycolate; PE, phosphatidyl ethanolamine; PG, phosphatidylglycerol; PI, phosphatidylinositol; PIM, phosphoinositolmannosides; PGL, phenolic glycolipid.
(TIF)

Figure S10 **Lipid analysis of the Δ** ***mmpS4/S5 M. tuberculosis*** **mutant by two-dimensional thin layer chromatography of apolar and polar lipids.** The profiles **A–G** show lipids of the apolar fraction, the profiles **H** and **I** are polar lipids. TLC plates A, B, C, F, G, H, I show lipids corresponding to 2 mg of dry delipidated cells, while plates D and E were loaded with lipids corresponding to 1 mg of dry delipidated cells. Profiles A–C were resolved using solvent system a. Profiles D and E were resolved using solvent system e. Profiles F–I were resolved using solvent system F. All TLCs were sprayed with Rhodamine G6 to visualize neutral lipids and phospholipids. MK, menaquinone; TG, triacylglycerols; J and K, apolar mycolipenates of trehalose; L, free fatty acids; SL, sulfathides (sulfolipids); DAT, 2,3-di-O-acyltrehalose; WE, waxy esters; ?, unknown.
(TIF)

Figure S11 **Southern blot analysis of triple deletion mutants in avirulent** ***M. tuberculosis*** **.** Chromosomal DNA of *Mtb* strains were digested with BamHI and ApaI for analysis of *mmpS4/mmpL4* and *mmpS5/mmPL5* genomic regions, respectively. Digested chromosomal DNA was analyzed by Southern blotting using probes generated by PCR from genomic DNA. In-frame deletions of the *mmpS4/mmpL4* and the *mmpS5/mmPL5* operons were constructed by homologous recombination and subsequent excision of the hygromycin resistance marker by Cre recombinase.
(TIF)

Figure S12 **Predicted membrane topologies of MmpS4 and MmpL4.** The figure was drawn according to the prediction results of the Mobyle@Pasteur server (http://mobyle.pasteur.fr/cgi-bin/portal.py#forms::toppred).
(TIF)

Figure S13 **Gross pathology of mouse lungs infected with** ***M. tuberculosis*** **.** Gross pathology of whole lungs of BALB/c mice infected with wt *Mtb* H37Rv (ML617), Δ*mmpS4/S5* (ML618), Δ*mmpS4/S5* singly complemented with *mmpS5* (ML619), Δ*mmpS4/S5* singly complemented with *mmpS4* (ML620), or Δ*mmpS4/S5* fully complemented with *mmpS4* and *mmpS5* (ML624).
(TIF)

Figure S14 **Effect of** ***mmpS4*** **and** ***mmpS5*** **on the pathology of mouse lungs infected with** ***Mtb*** **.** Lung sections of BALB/c mice infected with wt *Mtb* H37Rv (ML617), Δ*mmpS4/S5* (ML618), Δ*mmpS4/S5* singly complemented with *mmpS5* (ML619), Δ*mmpS4/S5* singly complemented with *mmpS4* (ML620), or Δ*mmpS4/S5* fully complemented with *mmpS4* and *mmpS5* (ML624). Pictures were taken with a Nikon Eclipse E800 microscope outfitted with a Nikon DXM1200 digital camera (magnification: 20×).
(TIF)

Figure S15 **Comparison of the structures of MmpS4 of** ***M. tuberculosis*** **and a putative calcium-binding protein of** ***Parabacteroides distasonis*** **.** **A.** Alignment of the three-dimensional structures of MmpS4 of *M. tuberculosis* (green) and a putative calcium-binding protein YP_001302112.1 of *Parabacteroides distasonis* (cyan). **B.** Comparison of topologies of MmpS4 (left) and YP_001302112.1 (right). Red triangles represent β-strands and blue circles represent α helices. The triangle of MmpS4 representing a putative eighth β strand is transparent because it is not visible in the structure.
(TIF)

Figure S16 **Purification of MmpS4_52–140_ by size-exclusion chromatography.** A Superdex 75 10/300 GL column (GE Healthcare) was used. The monomer fraction had a retention volume of about 14 ml.
(TIF)

Figure S17 **^1^H–^15^N HSQC spectra of MmpS4_52–140_.** The spectra were recorded at 298 K. The resonance assignments are indicated by one-letter amino acid codes and residue numbers.
(TIF)

Figure S18 **Chemical shifts of Cα and Cβ of MmpS4_52–140_ were assigned through HNCACB and CBCA(CO)NH spectra.**
(TIF)

Figure S19 **Chemical shift differences for CO, Cα and Cβ with respect to the chemical shift values of residues in random coil conformation and secondary structure of MmpS4_52–140_.** The secondary structure of MmpS4 was derived using the TALOS+ software. Positive values indicate β-sheets as secondary structures.
(TIF)

Table S1 **Strains used in this work.** The annotations hyg^R^ and kan^R^ indicate that the strain is resistant to the antibiotics hygromycin and kanamycin, respectively. Mutant strains were constructed from both the avirulent *Mtb* mc^2^6230 strain and the virulent *Mtb* H37Rv strain as indicated.
(DOCX)

Table S2 **Oligonucleotides used in this work.** Restriction sites used for cloning are underlined.^ a^SpeI, ^b^PacI, ^c^BfrBI, ^d^HindIII, ^e^NdeI
(DOCX)

Table S3 **Plasmids used in this work.** Up- and downstream homologous sequences of the *mmpS5* and *mmpS4* genes are subscripted as up and down in pML1500 and pML1501 for *mmpS5* and in pML1508 and pML1509 for *mmpS4*. “Origin” means origin of replication. The genes *bla*, *hyg* and *aph* confer resistance to ampicillin, hygromycin and kanamycin, respectively. The L5 and Ms6 *attP* sites are required for site specific integration of plasmids into the chromosomal L5 and Ms6 *attB* sites by the mycobacteriophage L5 and Ms6 integrase *int* genes, respectively. The site-specific recombinase Cre excises DNA fragments that are flanked by *loxP* recognition sites. pAL5000ts denotes the temperature-sensitive origin of replication of the pAL5000 plasmid. *E. coli* codon optimized *mmpS4* and *mmpS5* are designated *mmpS4_e_* and *mmpS5_e_*, respectively. The truncated *E. coli* codon optimized gene of *mmpS4* encoding an N-terminal 6xhis tag is designated *his-mmpS4_24–140_*. The truncated *E. coli* codon optimized gene of *mmpS5* encoding an N-terminal 6xhis tag is designated *his-mmpS5_27–142_*. The native promoters of *mmpS4* and *mmpS5* are designated *p_native_-mmpS4* and *p_native_-mmpS5*, respectively, and include approximately 500 bp of their respective upstream regions. The *sacS* gene of *B. subtilis* encodes the counterselective marker levansucrase that mediates sensitivity to sucrose. Its expression is regulated by SacR.
(DOCX)

Table S4 **Statistics of the 20 lowest-energy conformer ensemble of MmpS4_52–140_.**
(DOCX)

Text S1 **Supplementary materials and methods.** Chemicals, enzymes, and DNA. Bacterial strains, media, and growth conditions. Plasmid construction. Construction of mutants in *Mtb* H37Rv and *Mtb* mc^2^6230. Complementation of *Mtb* mutants. Preparation and analysis of protein extracts from *M. tuberculosis*. Southern blot analysis of *M. tuberculosis*. Protein overexpression, purification, and antibody production. Growth rescue experiments of *M. tuberculosis* mutants with iron utilization defects. Extraction and analysis of *M. tuberculosis* lipids. Subcellular fractionation of *M. tuberculosis*. Cloning, expression and purification of MmpS4_52–140_. NMR spectroscopy. Assignment and data deposition. NMR Paramagnetic Relaxation Enhancement-Based Distance Measurements. Structure calculations. Interaction of MmpS4 and MmpL4.
(DOCX)
