# Supplementary figures and images for: Discovery of a Siderophore Export System Essential for Virulence of Mycobacterium tuberculosis
Source: PLoS Pathog. 2013 Jan 31;9(1):e1003120. doi: 10.1371/journal.ppat.1003120 (PMC3561183; doi:10.1371/journal.ppat.1003120)

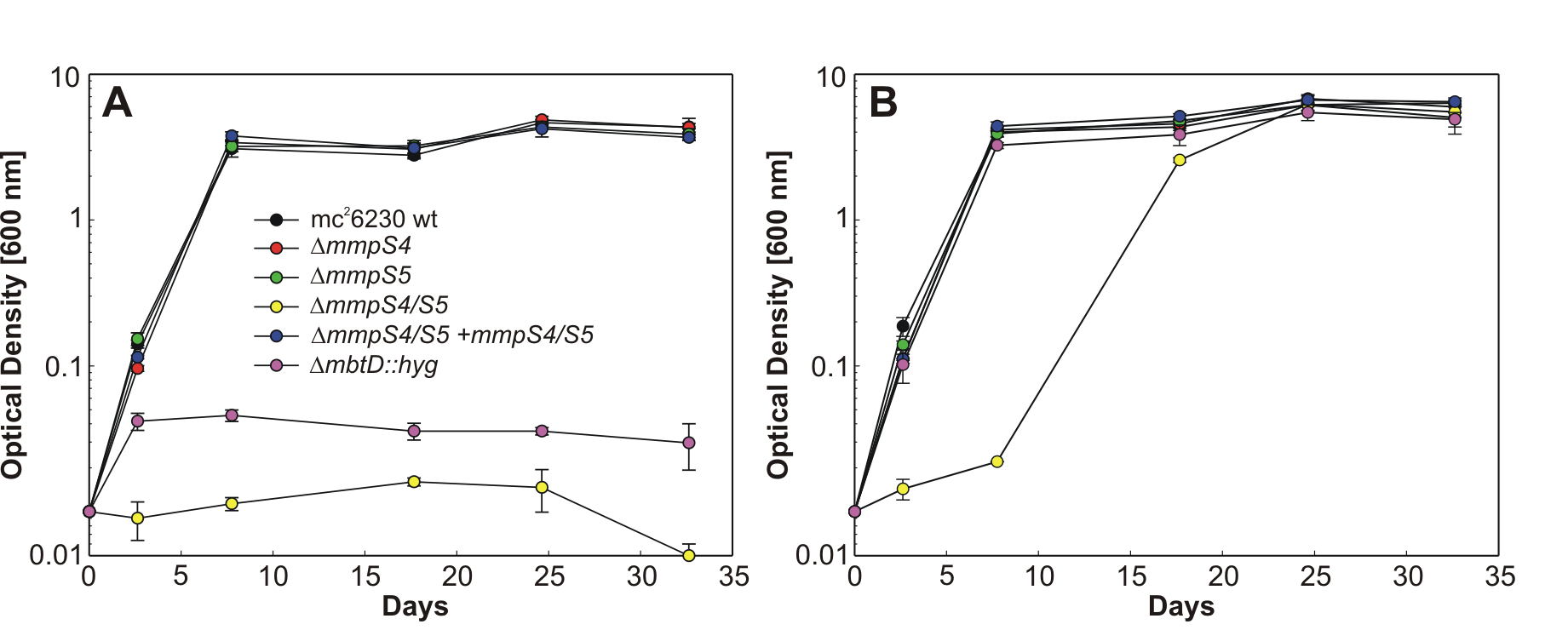

Supplement: Figure S1 — Growth of Mtb in low iron media. Growth of wt Mtb, ΔmmpS4, ΔmmpS5, ΔmmpS4/S5, fully complemented ΔmmpS4/S5 (+mmpS4/S5) and ΔmbtD::hyg in low iron HdB media supplemented with 10% OADC, 0.2% casamino acids, 24 µg/ml pantothenate, and 0.02% tyloxapol without (A) and with (B) 20 µM hemin as an iron source. (TIF) [file ppat.1003120.s001.tif]

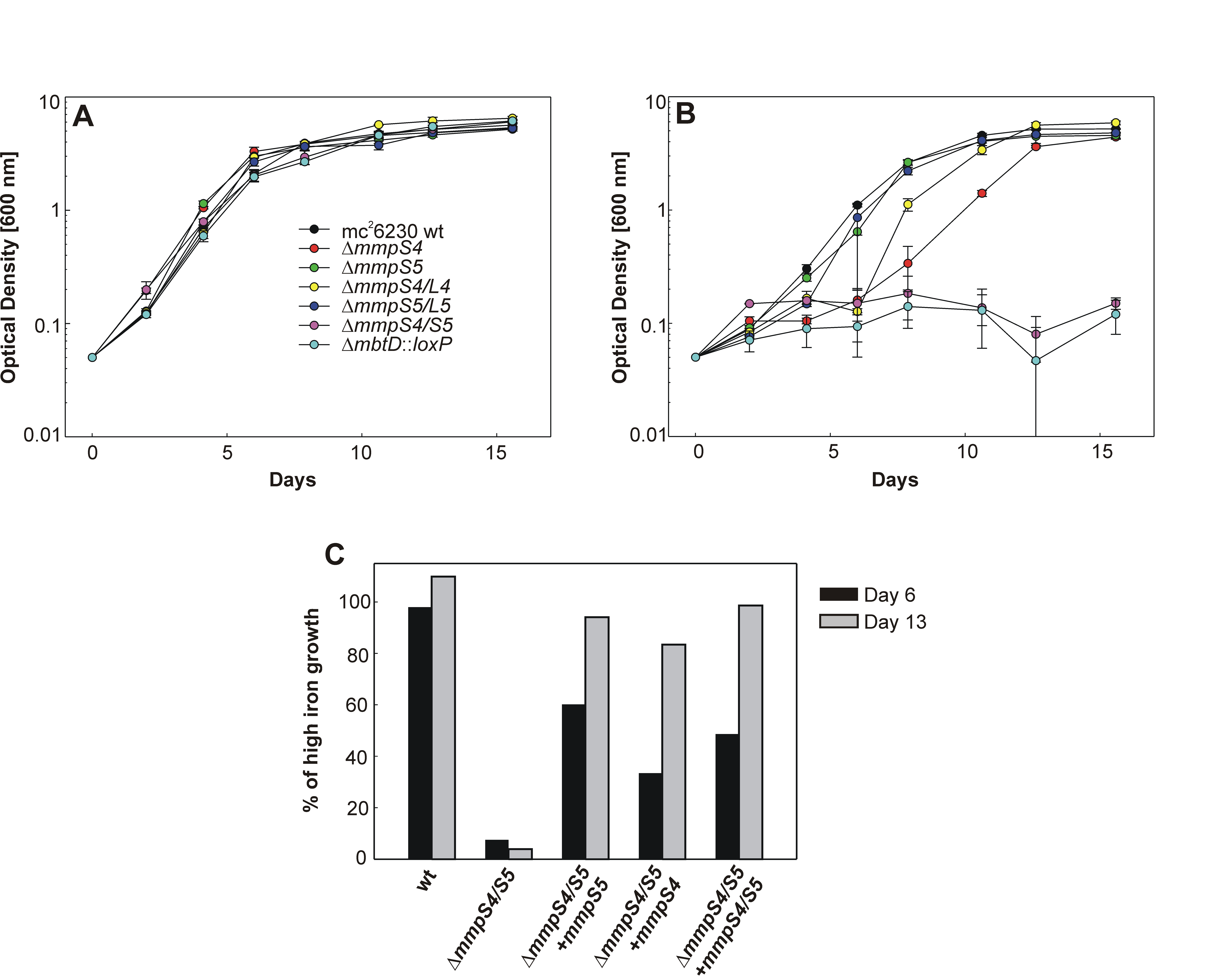

Supplement: Figure S2 — Growth of Mtb in 7H9 media supplemented with 2,2′-dipyridyl. A. Growth of wt Mtb, ΔmmpS4, ΔmmpS5, ΔmmpS4/L4, ΔmmpS5/L5, ΔmmpS4/S5, and ΔmbtD:: loxP strains in iron-replete 7H9 media supplemented with 10% OADC, 0.2% casamino acid, 24 µg/ml pantothenate, and 0.01% tyloxapol. Strains were started out at an OD600 = 0.05 and optical densities were measured at regular intervals for 15 days. Strains were grown in triplicate and standard deviations are shown. B. Growth of wt Mtb, ΔmmpS4, ΔmmpS5, ΔmmpS4/L4, ΔmmpS5/L5, ΔmmpS4/S5, and ΔmbtD::loxP strains in iron-deplete 7H9 media supplemented with 10% OADC, 0.2% casamino acids, 24 µg/ml pantothenate, 0.01% tyloxapol, and 0.1 mM 2,2′-dipyridyl (DIP) as an iron chelator. Strains were grown in triplicate and standard deviations are shown. C. Either mmpS4 or mmpS5 rescues the low iron delayed growth phenotype of ΔmmpS4/S5. Low iron growth in the presence of 0.1 mM DIP is reported as the percentage of growth in iron-replete media. Wild-type Mtb (ML617), ΔmmpS4/S5 (ML618), mmpS5 singly complemented ΔmmpS4/S5 (ML619), mmpS4 singly complemented ΔmmpS4/S5 (ML620), and ΔmmpS4/S5 fully complemented with mmpS4 and mmpS5 (ML624), were grown in both iron-replete 7H9 media and iron-deplete 7H9 supplemented with DIP. (TIF) [file ppat.1003120.s002.tif]

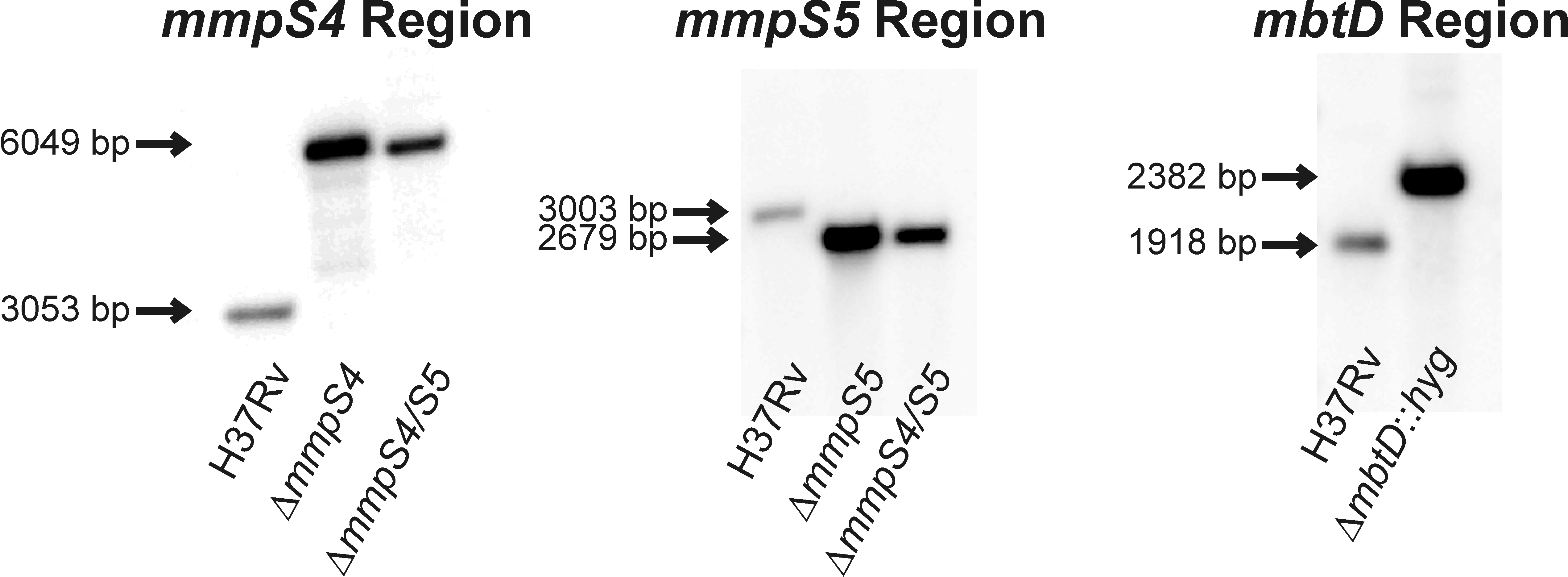

Supplement: Figure S3 — Southern blot analysis of deletion mutants in virulent Mtb . Chromosomal DNA of Mtb strains were digested with AatII, ApaI, or NruI, for analysis of mmpS4, mmpS5 and mbtD genomic regions, respectively. Digested chromosomal DNA was analyzed by Southern blotting using probes generated by PCR from genomic DNA. In frame deletions of mmpS4 and mmpS5 were constructed through homologous recombination and subsequent excision by Cre recombinase of the hyg marker. In the mmpS4 and mmpS5 deletion mutants, 373 and 363 bp, respectively, were replaced by loxP sites such that no stop codons were introduced into the reading frames. ML617 is the Mtb H37Rv parent wt strain. ML472 is the ΔmmpS4 single deletion strain. ML405 is the ΔmmpS5 single deletion strain. ML618 is the ΔmmpS4/S5 double deletion strain. ML1424 is the ΔmbtD::hyg deletion strain. (TIF) [file ppat.1003120.s003.tif]

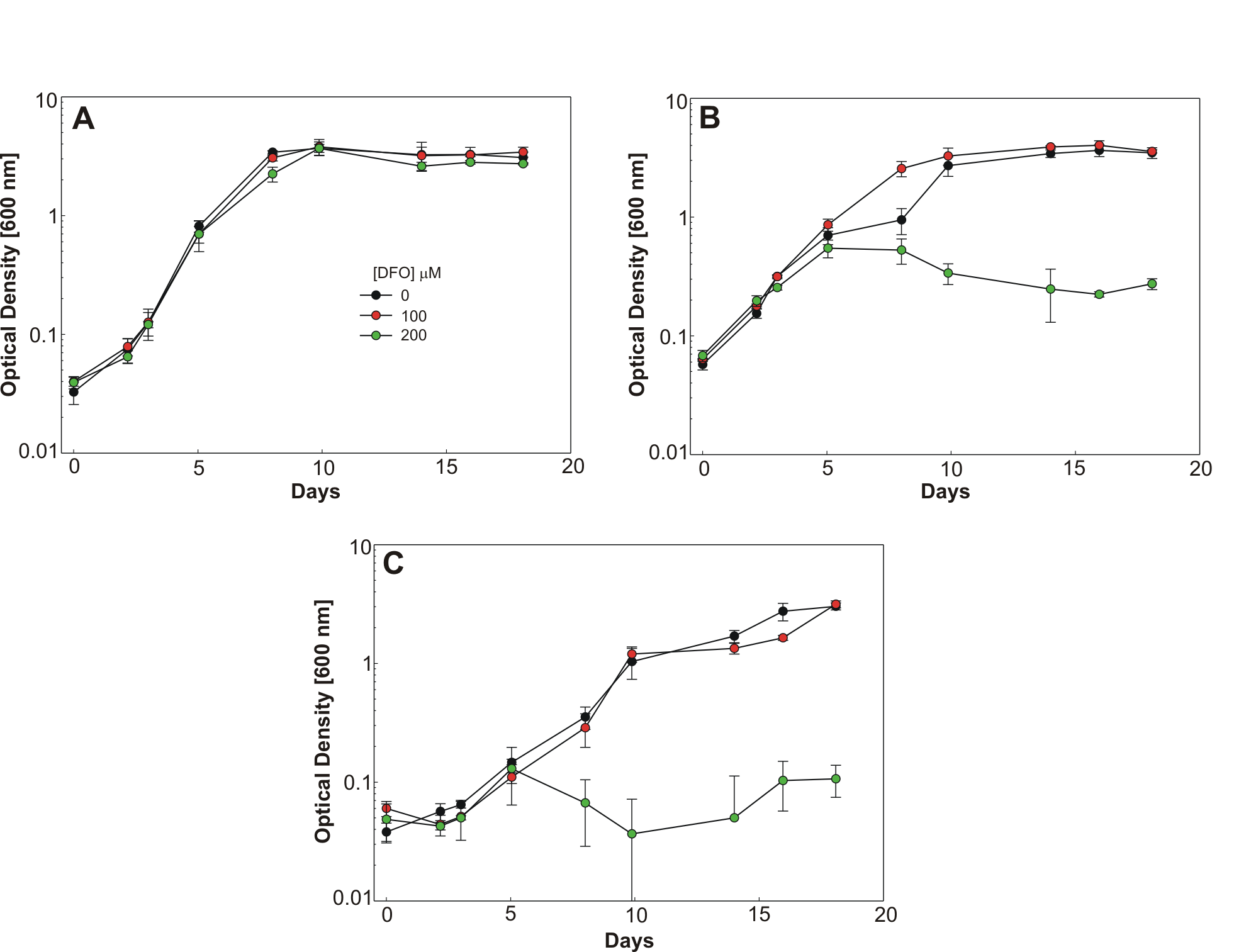

Supplement: Figure S5 — Growth of Mtb in 7H9 media containing desferrioxamine. Growth of wt Mtb (A), ΔmbtD::loxP (B) and ΔmmpS4/S5 (C) in 7H9 media supplemented with 10% OADC, 0.2% casamino acids and 24 µg/ml pantothenate containing 0 (black), 100 (red), or 200 µM (green) concentrations of the ferric specific chelator desferrioxamine (DFO). Strains were started out at an OD600 = 0.05 and optical densities were measured at regular intervals for 17 days. Strains were grown in triplicate and standard deviations are shown. (TIF) [file ppat.1003120.s005.tif]

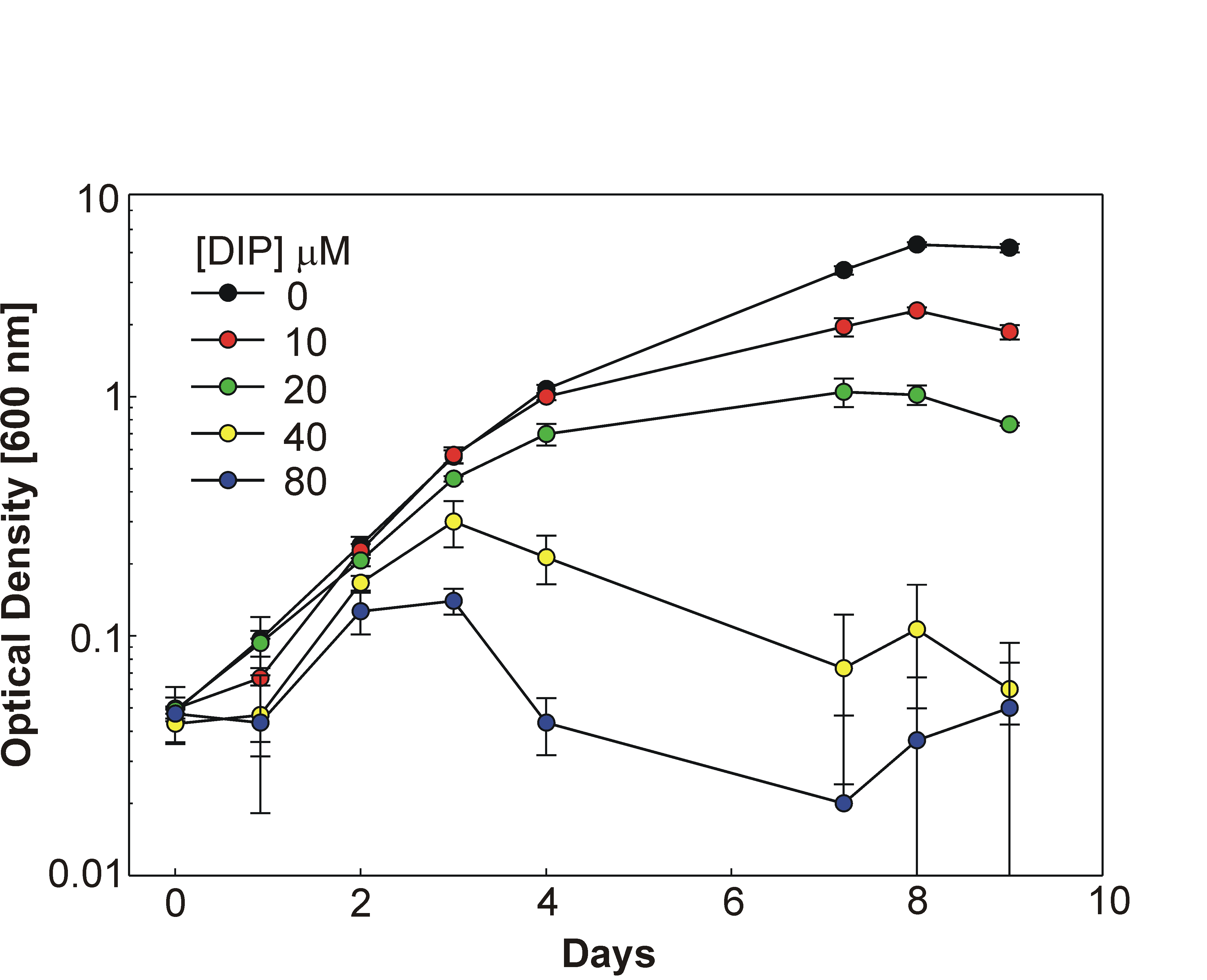

Supplement: Figure S6 — Growth of Δ mmpS4/S5 in 7H9 media containing 2,2′-dipyridyl. Growth of ΔmmpS4/S5 in 7H9 media supplemented with 10% OADC, 0.2% casamino acids and 24 µg/ml pantothenate containing 0 (black), 10 (red), 20 (green), 40 (yellow), or 80 (blue) µM concentrations of 2,2′-dipyridyl. Strains were started out at an OD600 = 0.05 and optical densities were measured at regular intervals for 9 days. Strains were grown in triplicate and standard deviations are shown. (TIF) [file ppat.1003120.s006.tif]

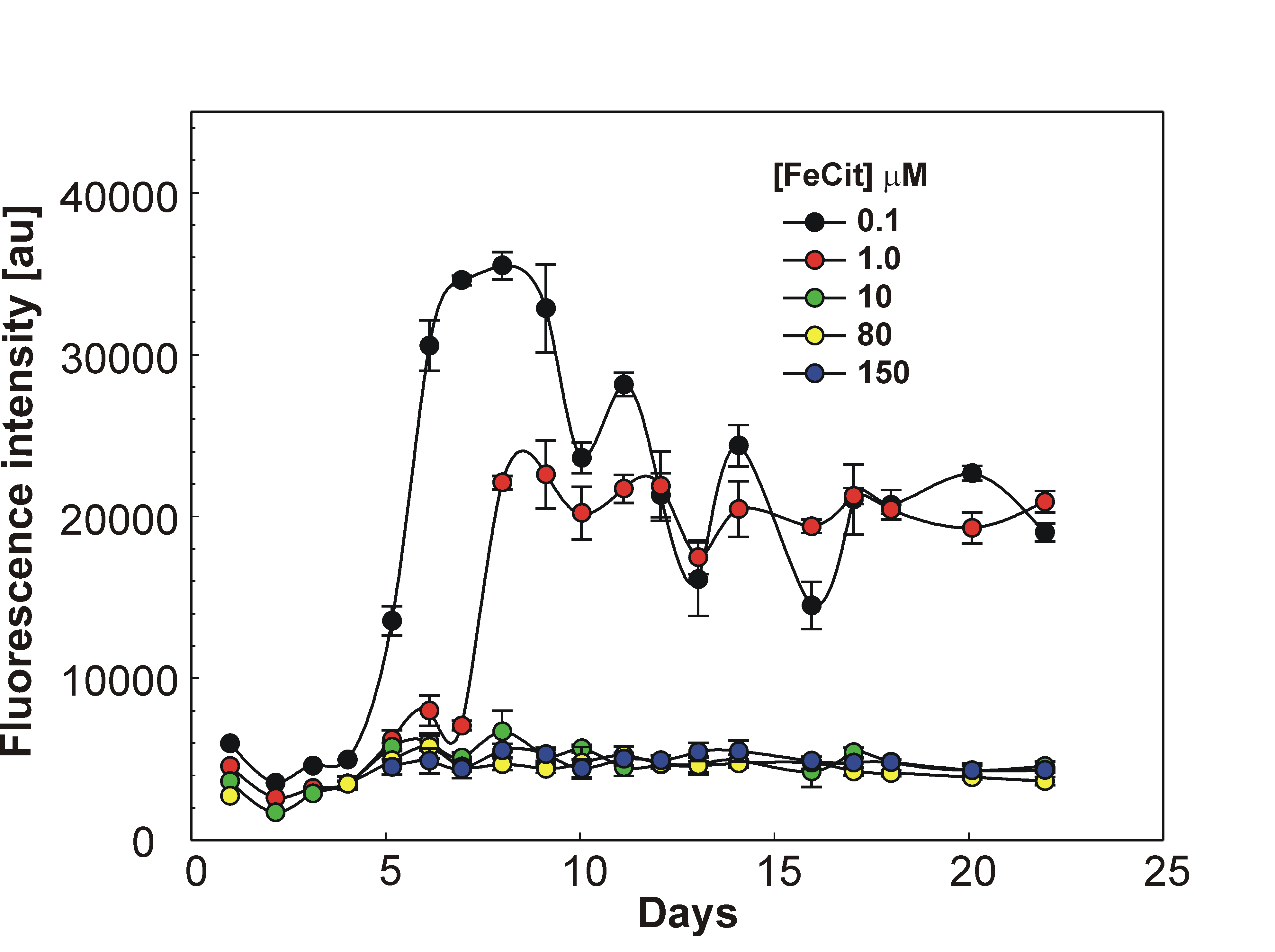

Supplement: Figure S7 — Validation of a cytoplasmic iron reporter in Mtb mc26230. Mtb mc26230 was grown in low iron HdB supplemented with 10% OADC, 0.2% casamino acids, 24 µg/ml pantothenate, 0.02% tyloxapol, and indicated ferric citrate concentrations. Fluorescence readings were taken at indicated time points as described in materials and methods. (TIF) [file ppat.1003120.s007.tif]

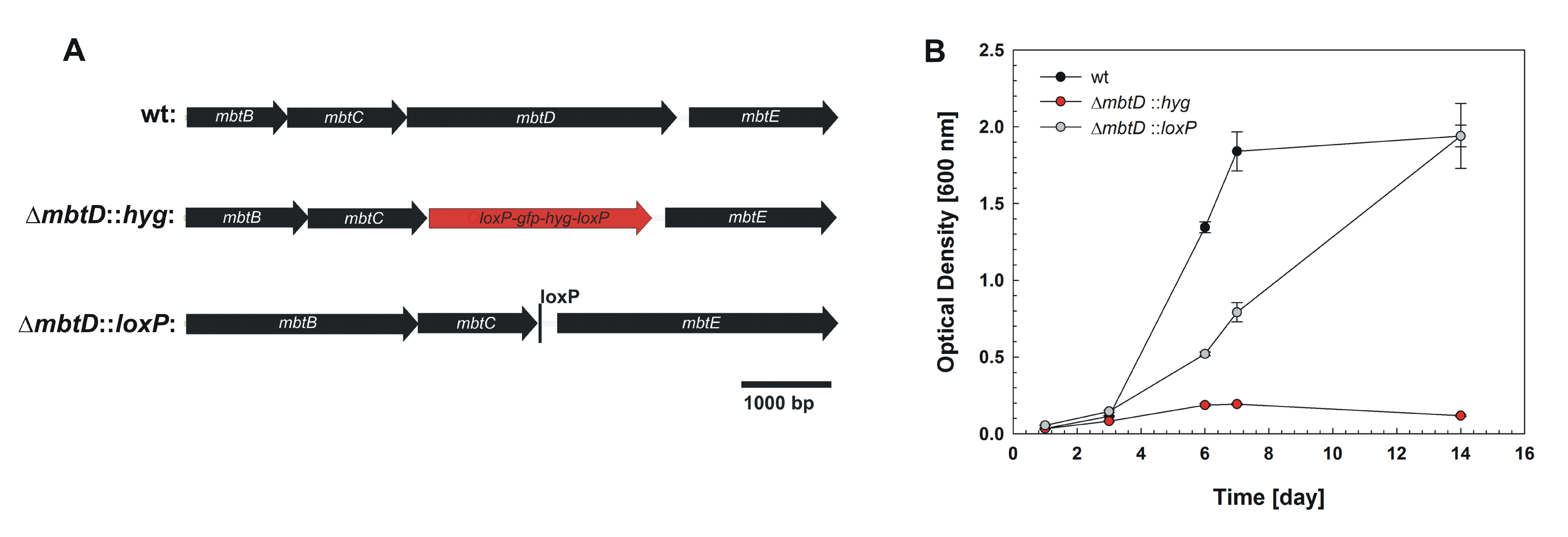

Supplement: Figure S8 — M. tuberculosis mbtD mutants used in this work. A. Genomic region encompassing mbtD in M. tuberculosis wt, ΔmbtD::hyg and ΔmbtD::loxP. B. Low iron growth phenotypes of wt, ΔmbtD::hyg, and ΔmbtD::loxP. Strains were grown in low iron HdB supplemented with 10% OADC, 0.2% casamino acids, 24 µg/ml pantothenate and 0.02% tyloxapol. (TIF) [file ppat.1003120.s008.tif]

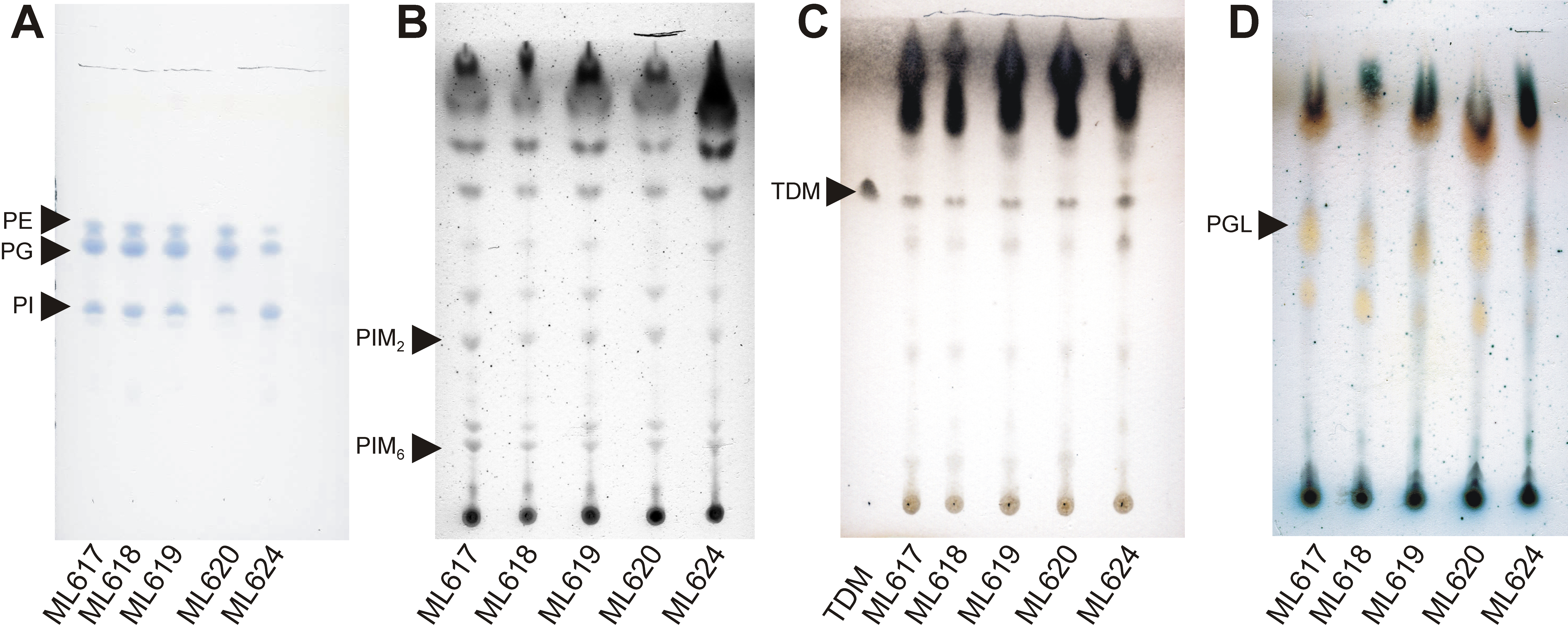

Supplement: Figure S9 — Lipid analysis of mmpS4 and mmpS5 mutants of M. tuberculosis by thin layer chromatography. Extracts of the indicated M. tuberculosis strains containing polar (A) and apolar (B, C, D) lipids were analyzed by thin layer chromatography (TLC). The amount of loaded lipids in A corresponds to 0.2 mg of delipidated cells, while the amounts loaded in B, C, D correspond to 0.4 mg. The TLC plates A and B were resolved using solvent system a. The TLC plate C was resolved using solvent system b and the plate D was resolved by solvent system c. Plate A shows phospholipids visualized by the Dittmer-Lester reagent. Anthrone was used to visualize sugar-containing lipids on plates B and D. Plate C shows trehalose mycolates visualized by copper sulfate in phosphoric acid. As a control, the first lane shows 4 mg of trehalose-di-mycolates (Sigma). TDM, trehalose dimycolate; PE, phosphatidyl ethanolamine; PG, phosphatidylglycerol; PI, phosphatidylinositol; PIM, phosphoinositolmannosides; PGL, phenolic glycolipid. (TIF) [file ppat.1003120.s009.tif]

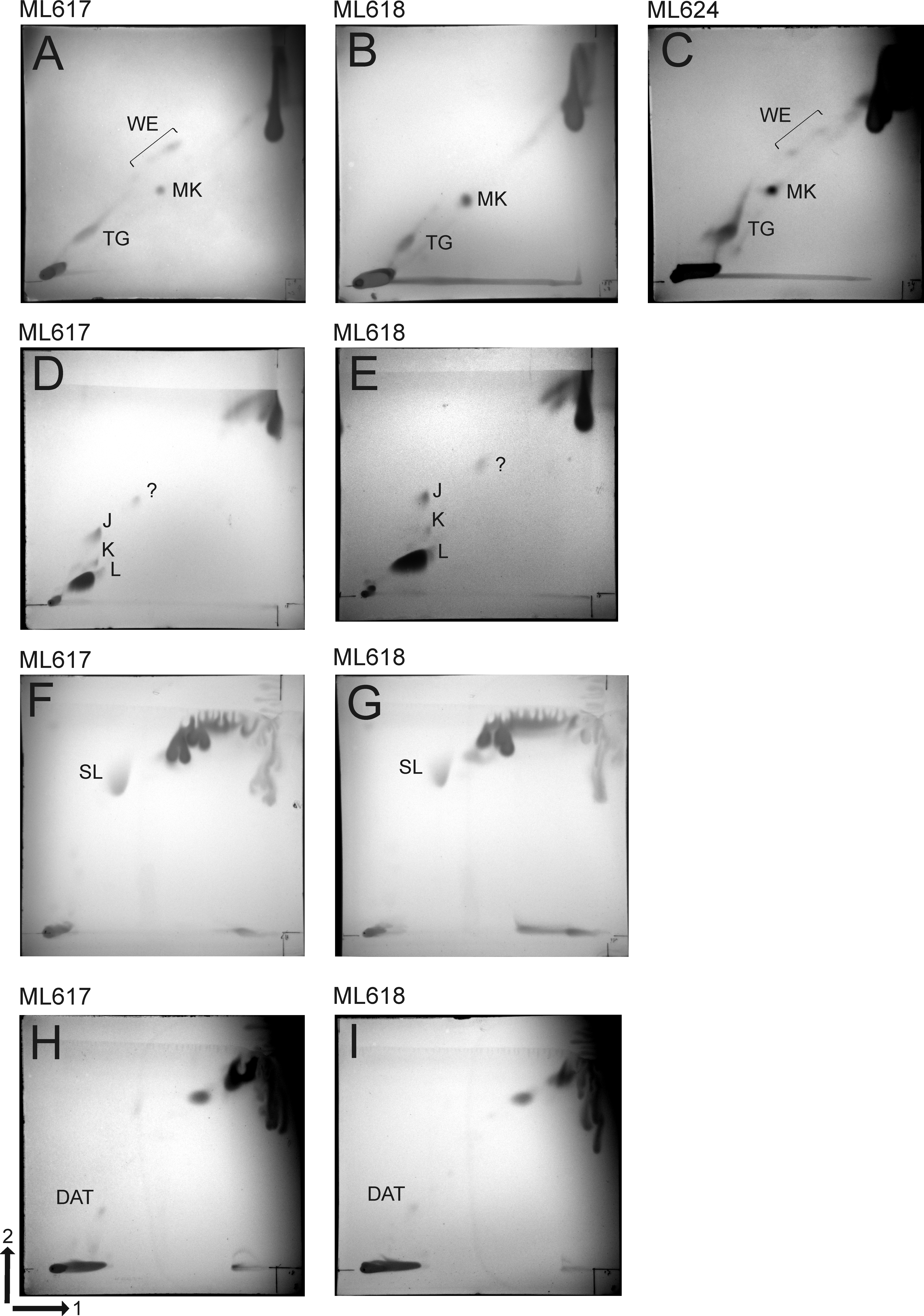

Supplement: Figure S10 — Lipid analysis of the Δ mmpS4/S5 M. tuberculosis mutant by two-dimensional thin layer chromatography of apolar and polar lipids. The profiles A–G show lipids of the apolar fraction, the profiles H and I are polar lipids. TLC plates A, B, C, F, G, H, I show lipids corresponding to 2 mg of dry delipidated cells, while plates D and E were loaded with lipids corresponding to 1 mg of dry delipidated cells. Profiles A–C were resolved using solvent system a. Profiles D and E were resolved using solvent system e. Profiles F–I were resolved using solvent system F. All TLCs were sprayed with Rhodamine G6 to visualize neutral lipids and phospholipids. MK, menaquinone; TG, triacylglycerols; J and K, apolar mycolipenates of trehalose; L, free fatty acids; SL, sulfathides (sulfolipids); DAT, 2,3-di-O-acyltrehalose; WE, waxy esters; ?, unknown. (TIF) [file ppat.1003120.s010.tif]

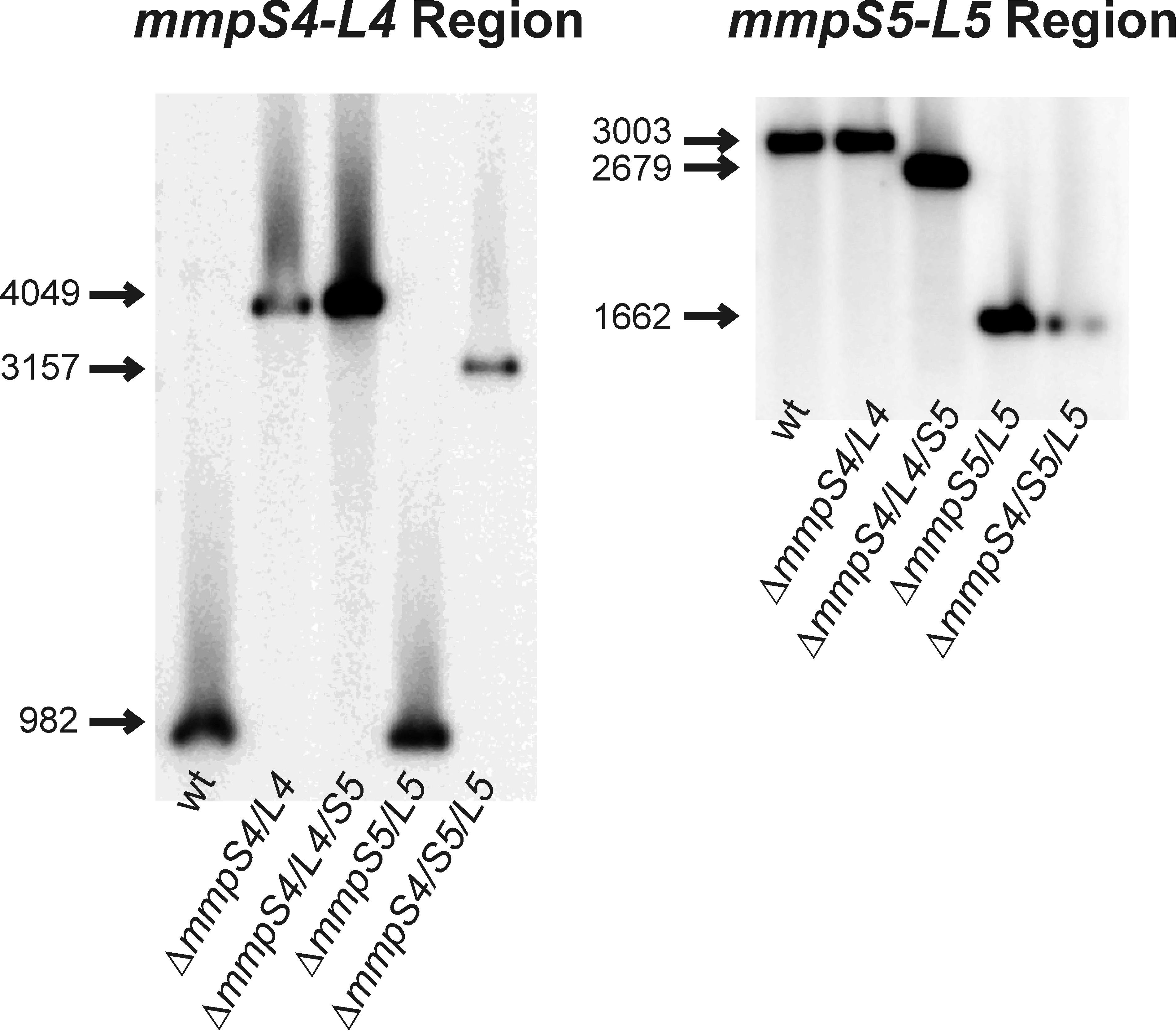

Supplement: Figure S11 — Southern blot analysis of triple deletion mutants in avirulent M. tuberculosis . Chromosomal DNA of Mtb strains were digested with BamHI and ApaI for analysis of mmpS4/mmpL4 and mmpS5/mmPL5 genomic regions, respectively. Digested chromosomal DNA was analyzed by Southern blotting using probes generated by PCR from genomic DNA. In-frame deletions of the mmpS4/mmpL4 and the mmpS5/mmPL5 operons were constructed by homologous recombination and subsequent excision of the hygromycin resistance marker by Cre recombinase. (TIF) [file ppat.1003120.s011.tif]

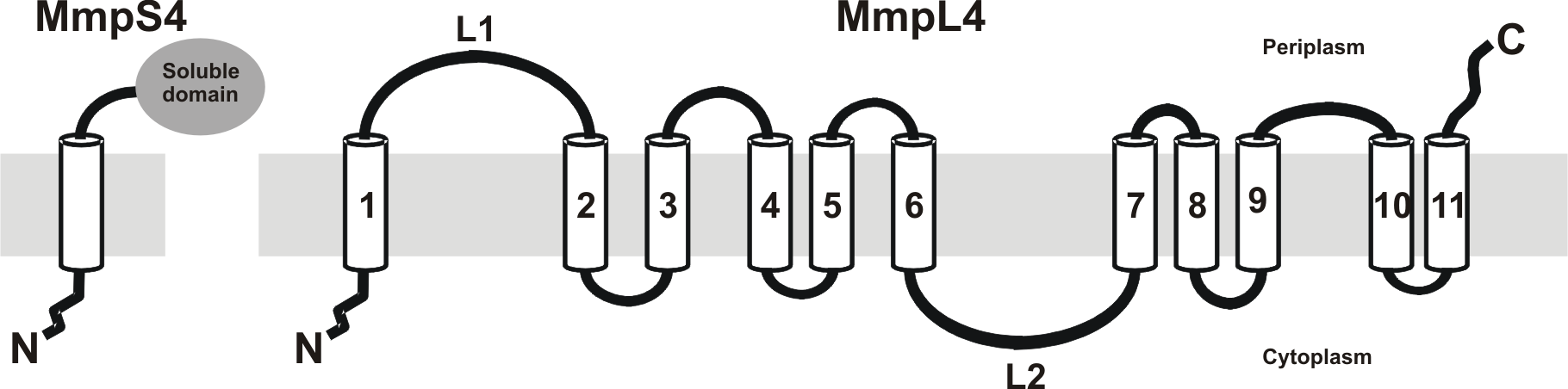

Supplement: Figure S12 — Predicted membrane topologies of MmpS4 and MmpL4. The figure was drawn according to the prediction results of the Mobyle@Pasteur server (http://mobyle.pasteur.fr/cgi-bin/portal.py#forms::toppred). (TIF) [file ppat.1003120.s012.tif]

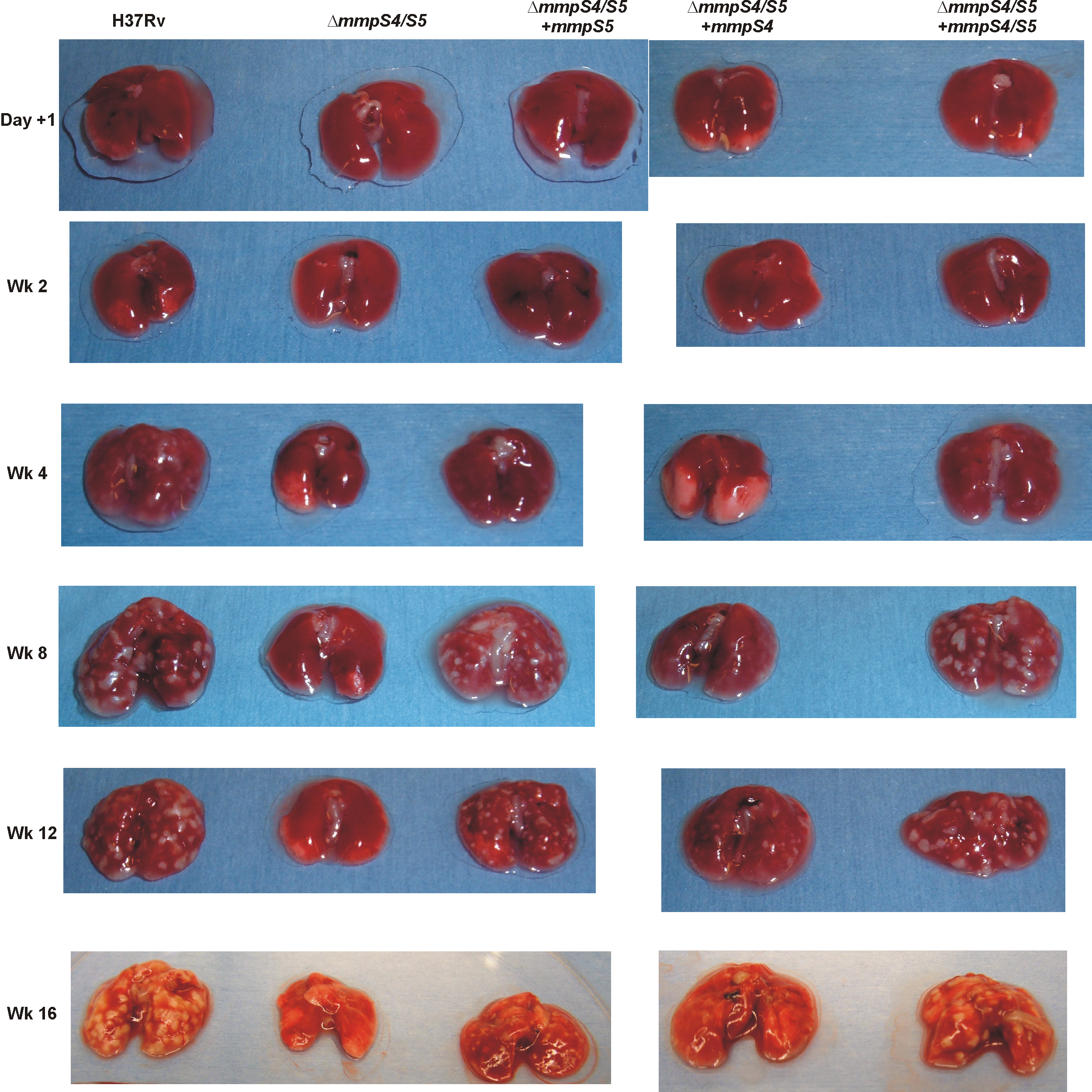

Supplement: Figure S13 — Gross pathology of mouse lungs infected with M. tuberculosis . Gross pathology of whole lungs of BALB/c mice infected with wt Mtb H37Rv (ML617), ΔmmpS4/S5 (ML618), ΔmmpS4/S5 singly complemented with mmpS5 (ML619), ΔmmpS4/S5 singly complemented with mmpS4 (ML620), or ΔmmpS4/S5 fully complemented with mmpS4 and mmpS5 (ML624). (TIF) [file ppat.1003120.s013.tif]

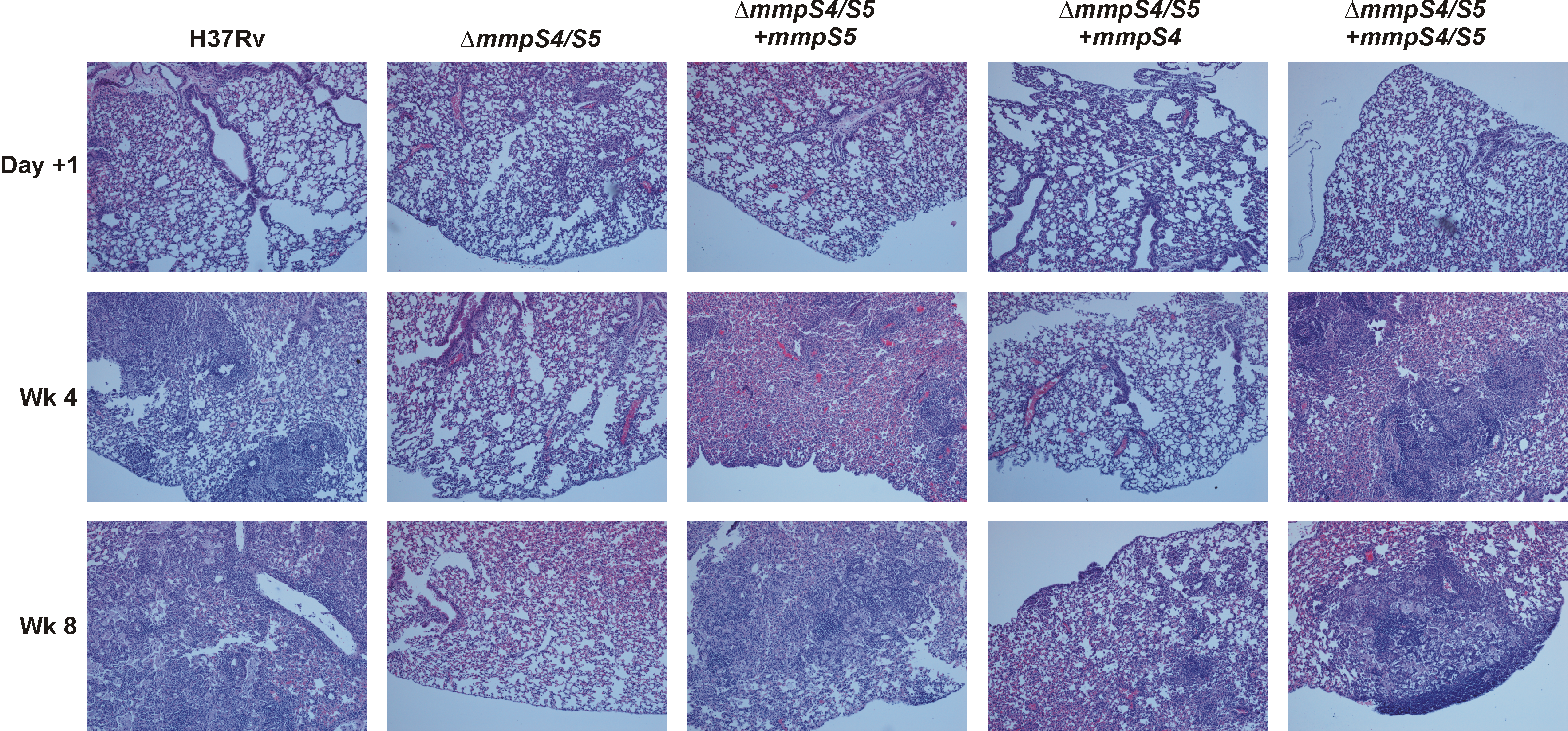

Supplement: Figure S14 — Effect of mmpS4 and mmpS5 on the pathology of mouse lungs infected with Mtb . Lung sections of BALB/c mice infected with wt Mtb H37Rv (ML617), ΔmmpS4/S5 (ML618), ΔmmpS4/S5 singly complemented with mmpS5 (ML619), ΔmmpS4/S5 singly complemented with mmpS4 (ML620), or ΔmmpS4/S5 fully complemented with mmpS4 and mmpS5 (ML624). Pictures were taken with a Nikon Eclipse E800 microscope outfitted with a Nikon DXM1200 digital camera (magnification: 20×). (TIF) [file ppat.1003120.s014.tif]

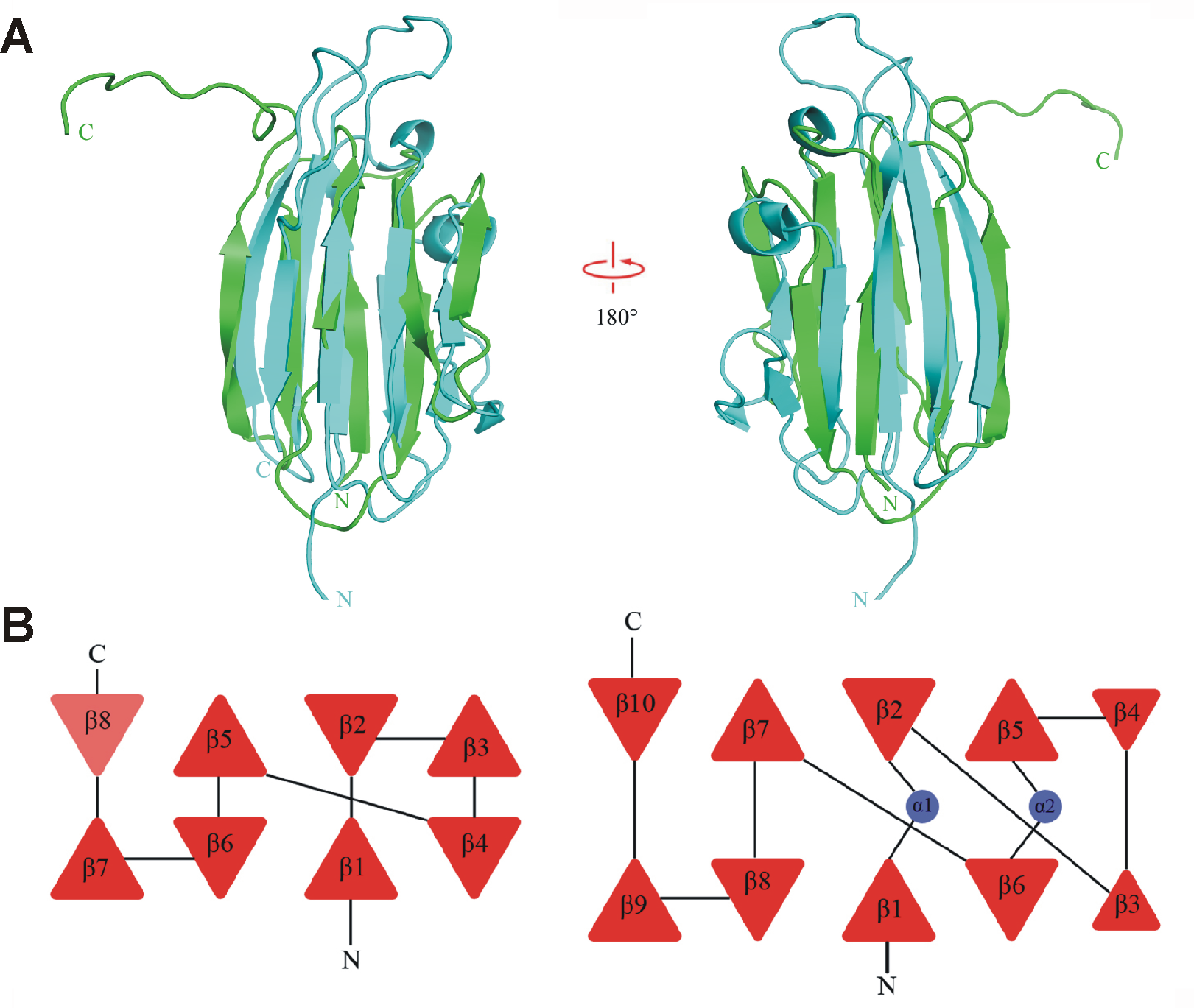

Supplement: Figure S15 — Comparison of the structures of MmpS4 of M. tuberculosis and a putative calcium-binding protein of Parabacteroides distasonis . A. Alignment of the three-dimensional structures of MmpS4 of M. tuberculosis (green) and a putative calcium-binding protein YP_001302112.1 of Parabacteroides distasonis (cyan). B. Comparison of topologies of MmpS4 (left) and YP_001302112.1 (right). Red triangles represent β-strands and blue circles represent α helices. The triangle of MmpS4 representing a putative eighth β strand is transparent because it is not visible in the structure. (TIF) [file ppat.1003120.s015.tif]

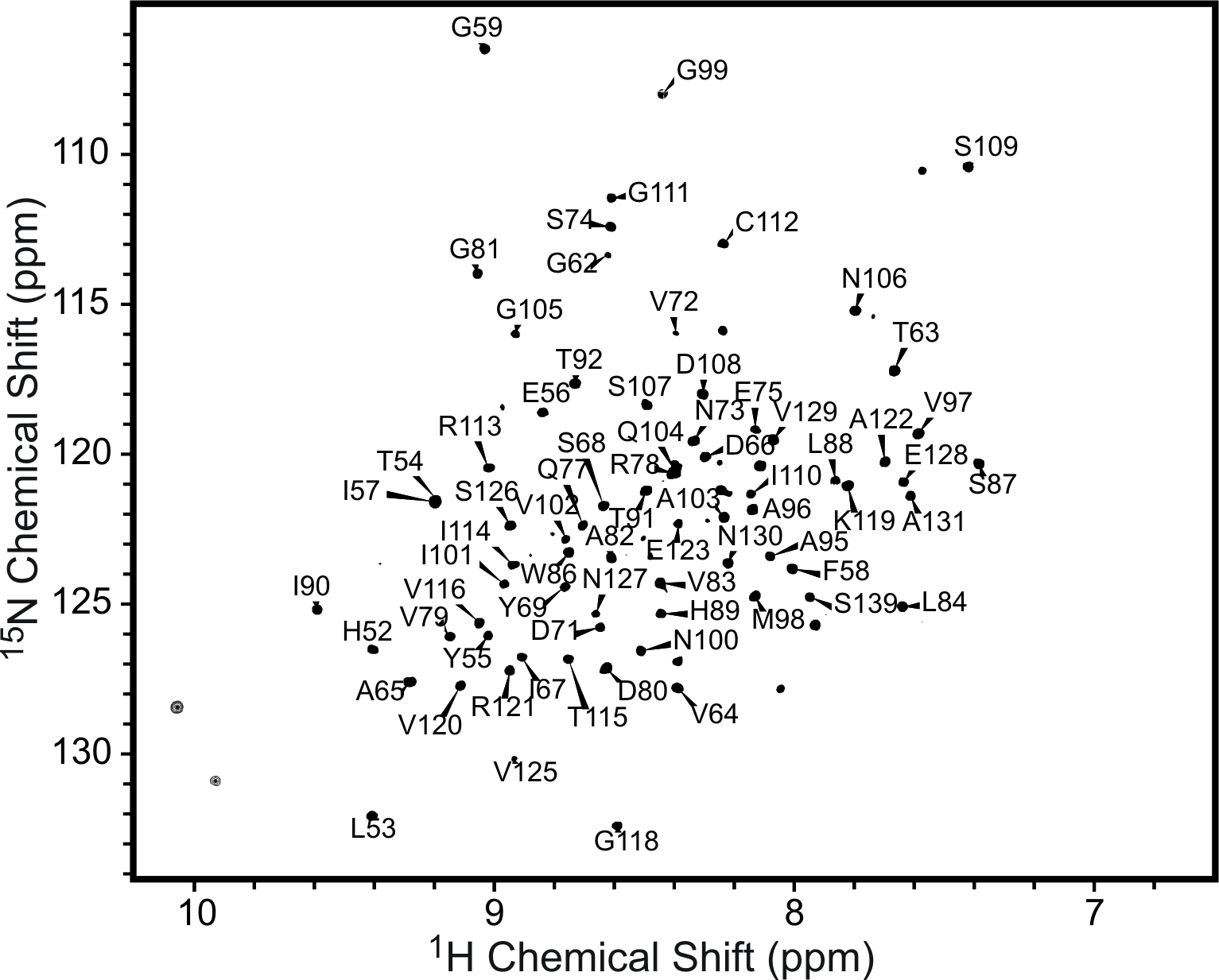

Supplement: Figure S17 — 1H–15N HSQC spectra of MmpS452–140. The spectra were recorded at 298 K. The resonance assignments are indicated by one-letter amino acid codes and residue numbers. (TIF) [file ppat.1003120.s017.tif]

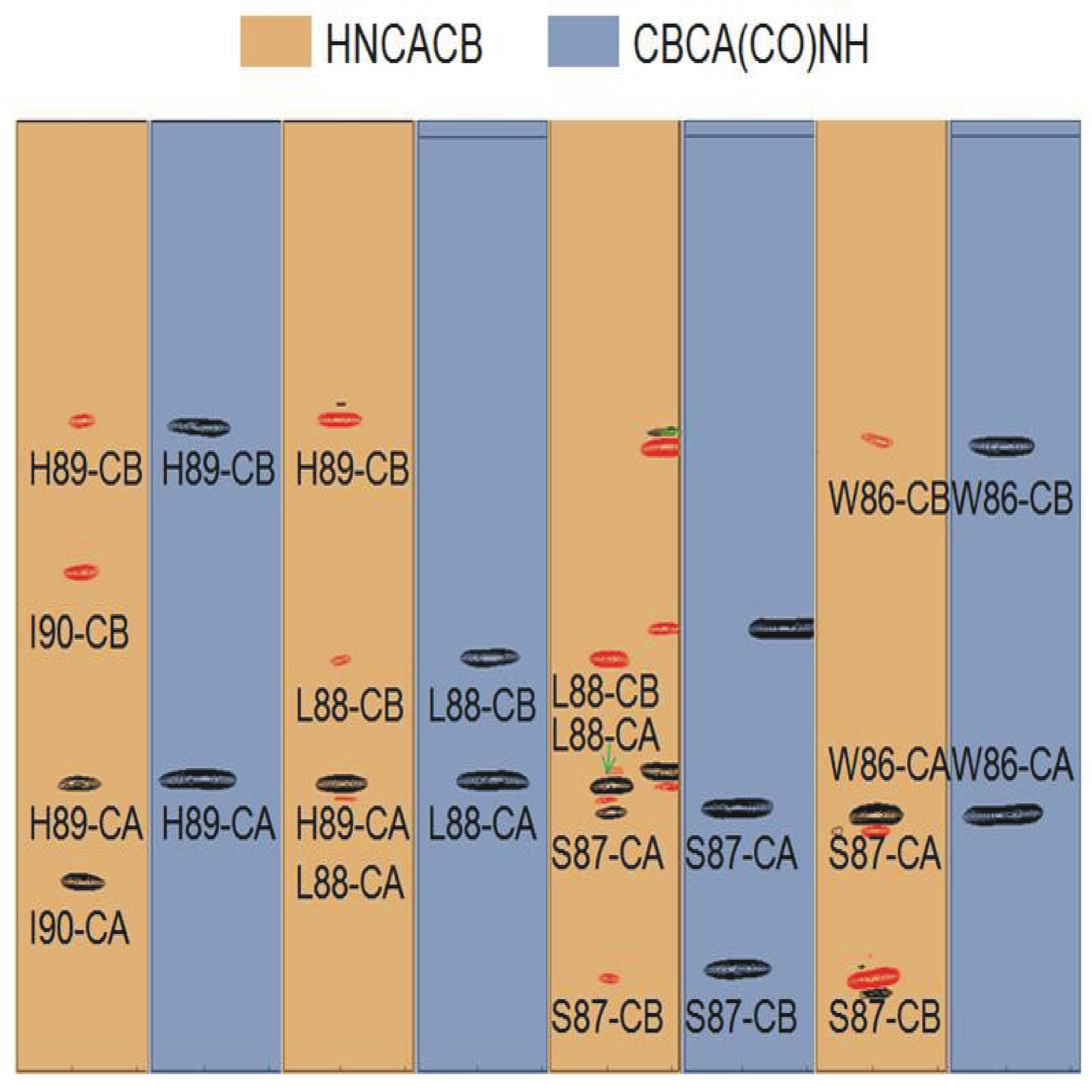

Supplement: Figure S18 — Chemical shifts of Cα and Cβ of MmpS452–140 were assigned through HNCACB and CBCA(CO)NH spectra. (TIF) [file ppat.1003120.s018.tif]

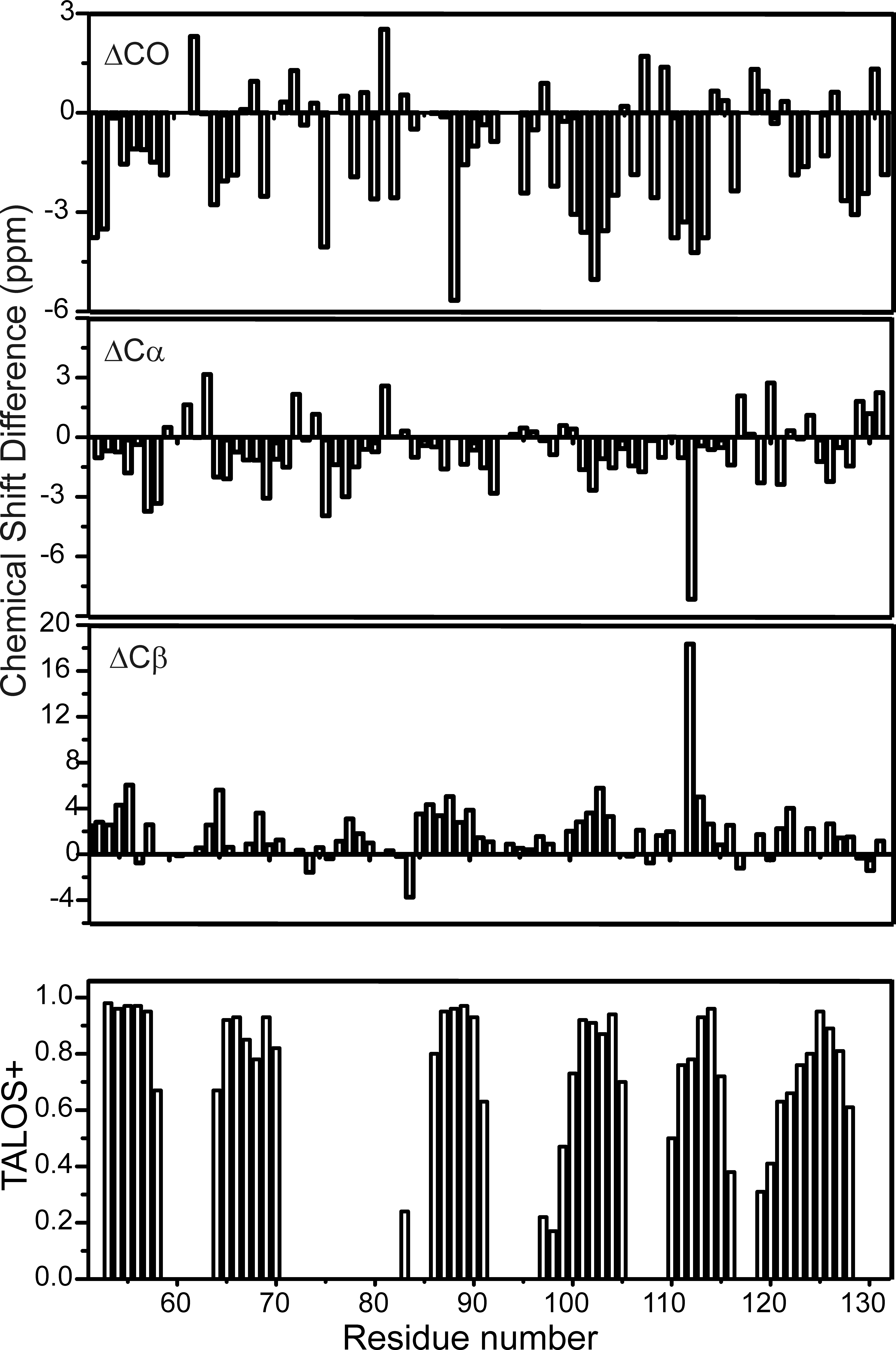

Supplement: Figure S19 — Chemical shift differences for CO, Cα and Cβ with respect to the chemical shift values of residues in random coil conformation and secondary structure of MmpS452–140. The secondary structure of MmpS4 was derived using the TALOS+ software. Positive values indicate β-sheets as secondary structures. (TIF) [file ppat.1003120.s019.tif]
