# Supplementary material for: Discovery of a Siderophore Export System Essential for Virulence of Mycobacterium tuberculosis
Source: PLoS Pathog. 2013 Jan 31;9(1):e1003120. doi: 10.1371/journal.ppat.1003120 (PMC3561183; doi:10.1371/journal.ppat.1003120)
Supplement: Table S1 — Strains used in this work. The annotations hygR and kanR indicate that the strain is resistant to the antibiotics hygromycin and kanamycin, respectively. Mutant strains were constructed from both the avirulent Mtb mc26230 strain and the virulent Mtb H37Rv strain as indicated. (DOCX) [file ppat.1003120.s020.docx]

| **Strain** | **Parent strain and relevant genotype** | **Source or reference** |
| --- | --- | --- |
| *E. coli* DH5α | *recA1; endA1; gyrA96; thi; relA1; hsdR17(rK-;mK+); supE44; φ80*Δ*lacZ*Δ*M15;* Δ*lacZYA-argF; UE169* | (1) |
| *M. tuberculosis* H37Rv | wild-type | ATCC# 25618 |
| *M. tuberculosis* mc^2^6230 | H37Rv derivative; Δ*RD1* Δ*panCD* | Dr. Jacobs (2) |
| *M. tuberculosis* ML405 | H37Rv derivative; Δ*mmpS5*::*loxP* | This study |
| *M. tuberculosis* ML472 | H37Rv derivative; Δ*mmpS4*::*loxP* | This study |
| *M. tuberculosis* ML482 | ML405 derivative; Δ*mmpS4*::*loxP,* Δ*mmpS5*::*loxP* | This study |
| *M. tuberculosis* ML617 | H37Rv derivative; L5 *attB*::pML1342, Ms6 *attB*::pML1562; hyg^R^, kan^R^ | This study |
| *M. tuberculosis* ML618 | ML482 derivative; Δ*mmpS4*::*loxP,* Δ*mmpS5*::*loxP*, L5 *attB*::pML1342, Ms6 *attB*::pML1562; hyg^R^, kan^R^ | This study |
| *M. tuberculosis* ML619 | ML482 derivative; Δ*mmpS4*::*loxP,* Δ*mmpS5*::*loxP*, L5 *attB*::pML1544, Ms6 *attB*::pML1562; hyg^R^, kan^R^ | This study |
| *M. tuberculosis* ML620 | ML482 derivative; Δ*mmpS4*::*loxP,* Δ*mmpS5*::*loxP*, L5 *attB*::pML1545, Ms6 *attB*::pML1562; hyg^R^, kan^R^ | This study |
| *M. tuberculosis* ML624 | ML482 derivative; Δ*mmpS4*::*loxP,* Δ*mmpS5*::*loxP*, L5 *attB*::pML1545, Ms6 *attB*::pML1560; hyg^R^, kan^R^ | This study |
| *M. tuberculosis* ML1424 | H37Rv derivative; Δ*mbtD*::*hyg* | This study |
| *M. tuberculosis* ML406 | mc^2^6230 derivative; Δ*mmpS5*::*loxP* | This study |
| *M. tuberculosis* ML475 | mc^2^6230 derivative; Δ*mmpS4*::*loxP* | This study |
| *M. tuberculosis* ML859 | ML406 derivative; Δ*mmpS4*::*loxP,* Δ*mmpS5*::*loxP* | This study |
| *M. tuberculosis* ML1451 | ML859 derivative; Δ*mmpS4*::*loxP,* Δ*mmpS5*::*loxP*, Δ*mbtD*::*hyg* | This study |
| *M. tuberculosis* ML878 | mc^2^6230 derivative; L5 *attB*::pML1342, Ms6 *attB*::pML1562; hyg^R^, kan^R^ | This study |
| *M. tuberculosis* ML1401 | ML859 derivative; Δ*mmpS4*::*loxP,* Δ*mmpS5*::*loxP,* L5 *attB*::pML1342, Ms6 *attB*::pML1562; hyg^R^, kan^R^ | This study |
| *M. tuberculosis* ML886 | ML859 derivative; Δ*mmpS4*::*loxP,* Δ*mmpS5*::*loxP,* L5 *attB*::pML1544, Ms6 *attB*::pML1562; hyg^R^, kan^R^ | This study |
| *M. tuberculosis* ML887 | ML859 derivative; Δ*mmpS4*::*loxP,* Δ*mmpS5*::*loxP,* L5 *attB*::pML1545, Ms6 *attB*::pML1562; hyg^R^, kan^R^ | This study |
| *M. tuberculosis* ML889 | ML859 derivative; Δ*mmpS4*::*loxP,* Δ*mmpS5*::*loxP,* L5 *attB*::pML1545, Ms6 *attB*::pML1560; hyg^R^, kan^R^ | This study |
| *M. tuberculosis* ML879 | mc^2^6230 derivative; L5 *attB*::pML1802, Ms6 *attB*::pML1562; hyg^R^, kan^R^ | This study |
| *M. tuberculosis* ML890 | ML859 derivative; Δ*mmpS4*::*loxP,* Δ*mmpS5*::*loxP,* L5 *attB*::pML1802, Ms6 *attB*::pML1562; hyg^R^, kan^R^ | This study |
| *M. tuberculosis* ML891 | ML859 derivative; Δ*mmpS4*::*loxP,* Δ*mmpS5*::*loxP,* L5 *attB*::pML1802, Ms6 *attB*::pML1560; hyg^R^, kan^R^ | This study |
| *M. tuberculosis* ML892 | ML859 derivative; Δ*mmpS4*::*loxP,* Δ*mmpS5*::*loxP,* L5 *attB*::pML1802, Ms6 *attB*::pML1561; hyg^R^, kan^R^ | This study |
| *M. tuberculosis* ML1600 | mc^2^6230 derivative; Δ*mbtD*::*hyg* | (3) |
| *M. tuberculosis* ML1610 | mc^2^6230 derivative; Δ*mbtD*::*loxP* | This study |
| *M. tuberculosis* ML1613 | ML1610 derivative; Δ*mbtD*::*loxP,* L5 *attB*::pML1802, Ms6 *attB*::pML1562; hyg^R^, kan^R^ | This study |
| *M. tuberculosis* ML1432 | ML406 derivative; Δ*mmpS4/mmpL4*::*loxP,* Δ*mmpS5*::*loxP* | This study |
| *M. tuberculosis* ML1433 | ML475 derivative; Δ*mmpS5/mmpL5*::*loxP,* Δ*mmpS4*::*loxP* | This study |
| *M. tuberculosis* ML1437 | ML1432 derivative; Δ*mmpS4/mmpL4*::*loxP,* Δ*mmpS5*::*loxP,* L5 a*ttB*::pML1544; hyg^R^ | This study |
| *M. tuberculosis* ML1438 | ML1432 derivative; Δ*mmpS4/mmpL4*::*loxP,* Δ*mmpS5*::*loxP,* L5 *attB*::pML1545; hyg^R^ | This study |
| *M. tuberculosis* ML1444 | ML1433 derivative; Δ*mmpS5/mmpL5*::*loxP,* Δ*mmpS4*::*loxP,* L5 *attB*::pML1342; hyg^R^ | This study |
| *M. tuberculosis* ML1445 | ML1433 derivative; Δ*mmpS5/mmpL5*::*loxP,* Δ*mmpS4*::*loxP,* L5 *attB*::pML1544; hyg^R^ | This study |
| *M. tuberculosis* ML1446 | ML1433 derivative; Δ*mmpS5/mmpL5*::*loxP,* Δ*mmpS4*::*loxP,* L5 *attB*::pML1545; hyg^R^ | This study |
| *M. tuberculosis* ML1452 | ML1432 derivative; Δ*mmpS4/mmpL4*::*loxP,* Δ*mmpS5*::*loxP,* L5 *attB*::pML1342; hyg^R^ | This study |

**Table S1. Strains used in this work.** The annotations hyg^R^ and kan^R^ indicate that the strain in resistant to the antibiotics hygromycin and kanamycin, respectively. Mutant strains were constructed from both the avirulent *Mtb* mc^2^6230 strain and the virulent *Mtb* H37Rv strain as indicated.
