# Supplementary material for: Discovery of a Siderophore Export System Essential for Virulence of Mycobacterium tuberculosis
Source: PLoS Pathog. 2013 Jan 31;9(1):e1003120. doi: 10.1371/journal.ppat.1003120 (PMC3561183; doi:10.1371/journal.ppat.1003120)
Supplement: Table S2 — Oligonucleotides used in this work. Restriction sites used for cloning are underlined. aSpeI, bPacI, cBfrBI, dHindIII, eNdeI (DOCX) [file ppat.1003120.s021.docx]

| **Oligonucleotide** | **Sequence (5’→3’)** |
| --- | --- |
| ^a^1409 | GCACTAGTGACCGCCACCACCACGACAG |
| 1410 | GTATCCAGGCACGCTTGAGAG |
| ^b^1411 | GCTTAATTAACCTGACCTACTGCTTTGTG |
| ^c^1412 | CATAGAATGCATCGATCAGGAAGATGGCGTAGTC |
| ^a^1568 | CGACTAGTCACCGCAGCCAGCAATGCCCCGAGTTC |
| 1569 | GCATTAGCACCTTGAAAATCTCAG |
| ^b^1570 | CGTTAATTAAAGTCAACGCCTATACTTACTGCTTG |
| ^c^1571 | GCATGCATGGTCAATCCAGAGCCCAGAATCACG |
| ^b^1767 | CGTTAATTAACCGGACCTTCGCCTTGGTCTGAGCCGTAC |
| ^c^1768 | GCATGCATCGATTTGTGGTGGCTGCGGCG |
| ^b^1773 | GCTTAATTAAGAGCAGCGACCGCCTCGGCTG |
| ^c^1774 | GCATGCATTAGCCTGCTGATGTCGTAGC |
| ^d^1811 | GCAAGCTTCGAATTTAGTACTCACGC |
| ^a^1812 | GCACTAGTGATCGGCGGCCAGACCGTTG |
| ^d^1813 | GCAAGCTTCGATCATGCGGATTTCACAAAGC |
| ^a^1814 | GCACTAGTGATCCCGCATCTCCCGCAGCC |
| 1953 | CGGAGCACTAGTGCTGTTATGTCGGTGCCT |
| 1954 | CGGACATTTAAATGGGCAGCTGTTTGGGGGC |
| 1984 | GATGCCTGGCAGTTTATG |
| ^e^2356 | GCCATATGCACCACCACCACCACCATCGTATCCGCGGCTTCTTTGGTAGC |
| ^e^2357 | GCCATATGCACCACCACCACCACCACCGTATCCGCACGTTTTTCGGCAGCGAAG |

**Table S2. Oligonucleotides used in this work.** Restriction sites used for cloning are underlined.

^a^SpeI, ^b^PacI, ^c^BfrBI, ^d^HindIII, ^e^NdeI
