# Supplementary material for: Discovery of a Siderophore Export System Essential for Virulence of Mycobacterium tuberculosis
Source: PLoS Pathog. 2013 Jan 31;9(1):e1003120. doi: 10.1371/journal.ppat.1003120 (PMC3561183; doi:10.1371/journal.ppat.1003120)
Supplement: Table S3 — Plasmids used in this work. Up- and downstream homologous sequences of the mmpS5 and mmpS4 genes are subscripted as up and down in pML1500 and pML1501 for mmpS5 and in pML1508 and pML1509 for mmpS4. “Origin” means origin of replication. The genes bla, hyg and aph confer resistance to ampicillin, hygromycin and kanamycin, respectively. The L5 and Ms6 attP sites are required for site specific integration of plasmids into the chromosomal L5 and Ms6 attB sites by the mycobacteriophage L5 and Ms6 integrase int genes, respectively. The site-specific recombinase Cre excises DNA fragments that are flanked by loxP recognition sites. pAL5000ts denotes the temperature-sensitive origin of replication of the pAL5000 plasmid. E. coli codon optimized mmpS4 and mmpS5 are designated mmpS4e and mmpS5e, respectively. The truncated E. coli codon optimized gene of mmpS4 encoding an N-terminal 6xhis tag is designated his-mmpS424–140. The truncated E. coli codon optimized gene of mmpS5 encoding an N-terminal 6xhis tag is designated his-mmpS527–142. The native promoters of mmpS4 and mmpS5 are designated pnative-mmpS4 and pnative-mmpS5, respectively, and include approximately 500 bp of their respective upstream regions. The sacS gene of B. subtilis encodes the counterselective marker levansucrase that mediates sensitivity to sucrose. Its expression is regulated by SacR. (DOCX) [file ppat.1003120.s022.docx]

| **Plasmid** | **Parent vector, relevant genotype and properties** | **Source or reference** |
| --- | --- | --- |
| pET-28b(+) | T7 promoter, transcription start and terminator, His-tag, *lacI*, *aph,* pBR322 ORI; 5368 bp | Novagen, WI |
| pMAL-c5X | pMB1 origin, *lacI*, *malE*, *bla*, Factor Xa cleavage site; 5677 bp | NEB, MA |
| pCreSacB1 | p*_groEL_*-*cre*, oriE, oriM, *sacR*, *sacB*, *aph*; 7891 bp | Dr. Steyn |
| pML523 | pUC origin, pAL5000ts origin; *sacB xylE loxP-gfp*^2+^_m_-*hyg*-*loxP*; 9,845 bp | (3) |
| pML1500 | pML523; *mmpS5*_up_ *loxP*-*gfp*^2+^_m_-*hyg*-*loxP*; 10,797 bp | This study |
| pML1501 | pML1500; *loxP*-*gfp*^2+^_m_-*hyg*-*loxP* *mmpS5*_down_; 11,662 bp | This study |
| pML1508 | pML523; *mmpS4*_up_ *loxP*-*gfp*^2+^_m_-*hyg*-*loxP*; 11,212 bp | This study |
| pML1509 | pML1508; *loxP*-*gfp*^2+^_m_-*hyg*-*loxP* *mmpS4*_down_; 12,175 bp | This study |
| pML1342 | ColE1 origin; L5 *int*, *hyg, xylE_m_ , loxP-*L5 *attP-lox*; *5,404 bp* | (4) |
| pML1544 | pML1342; *loxP-p_native_-mmpS5-*L5 *attP-loxP*; 6,308 bp | This study |
| pML1545 | pML1342; *loxP-p_native_-mmpS4-*L5 *attP-loxP*; 6,371 bp | This study |
| pML2300 | ColE1 origin*; xylE_m_, gfp_m_^2+^, aph*, Ms6 *int*, Ms6 *attP*; *6,821 bp* | (4) |
| pML1560 | ColE1 origin; *xylE_m_, aph*, *p_native_-mmpS5,* Ms6 *int*, Ms6 *attP*; *6,688 bp* | This study |
| pML1561 | ColE1 origin; *xylE_m_, aph*, *p_native_-mmpS4,* Ms6 *int*, Ms6 *attP*; *6,751 bp* | This study |
| pML1562 | ColE1 origin*, xylE_m_, aph*, Ms6 *int*, Ms6 *attP*; *5,746 bp* | This study |
| pML1565 | pML1500; *loxP*-*gfp*^2+^_m_-*hyg*-*loxP* *mmpL5*_down_; 11,780 bp | This study |
| pML1566 | pML1508; *loxP*-*gfp*^2+^_m_-*hyg*-*loxP* *mmpL4*_down_; 12,293 bp | This study |
| pML1570 | pMAL-c5X; *malE*- *mmpS5_e_*; 6,096 bp | This study |
| pML1571 | pMAL-c5X; *malE*- *mmpS4_e_*; 6,090 bp | This study |
| pML1595 | pET-28b(+); *his-mmpS5_27-142_*; 5,552 bp | This study |
| pML1596 | pET-28b(+); *his-mmps4_24-140_*; 5,559 bp | This study |
| pML1801 | ColE1 origin, pAL5000 origin; hyg^R^, *fxbA23*_*gfp*^2+^_m_; 6,164 bp | (5) |
| pML1802 | pML1342; *loxP-fxbA23_ gfp^2+^_m_*-L5 *attP-loxP*; 6,369 bp | This study |
| pML1816 | pML523; *mbtD*_up_ *loxP*-*gfp*^2+^_m_-*hyg-loxP mbtD*_down_; 11, 825 bp | (3) |
| pML1828 | ColE1 origin, pAL5000 origin; *hyg, mbtG, HA tag –HIS tag*; 6,853 bp | This study |

**Table S3. Plasmids used in this work.** Up- and downstream homologous sequences of the *mmpS5* and *mmpS4* genes are subscripted as up and down in pML1500 and pML1501 for *mmpS5* and in pML1508 and pML1509 for *mmpS4*. “Origin” means origin of replication. The genes *bla*, *hyg* and *aph* confer resistance to ampicillin, hygromycin and kanamycin, respectively. The L5 and Ms6 *attP* sites are required for site specific integration of plasmids into the chromosomal L5 and Ms6 *attB* sites by the mycobacteriophage L5 and Ms6 integrase *int* genes, respectively. The site-specific recombinase Cre excises DNA fragments that are flanked by *loxP* recognition sites. pAL5000ts denotes the temperature-sensitive origin of replication (6) of the pAL5000 plasmid (7). *E. coli* codon optimized *mmpS4* and *mmpS5* are designated *mmpS4_e_* and *mmpS5_e_*, respectively. The truncated *E. coli* codon optimized gene of *mmpS4* encoding an N-terminal 6xhis tag is designated *his-mmpS4_24-140_*. The truncated *E. coli* codon optimized gene of *mmpS5* encoding an N-terminal 6xhis tag is designated *his-mmpS5_27-142_*. The native promoters of *mmpS4* and *mmpS5* are designated *p_native_-mmpS4* and *p_native_-mmpS5*, respectively, and include approximately 500 bp of their respective upstream regions. The *sacS* gene of *B. subtilis* encodes the counterselective marker levansucrase that mediates sensitivity to sucrose (8). Its expression is regulated by SacR.
