# Supplementary material for: Discovery of a Siderophore Export System Essential for Virulence of Mycobacterium tuberculosis
Source: PLoS Pathog. 2013 Jan 31;9(1):e1003120. doi: 10.1371/journal.ppat.1003120 (PMC3561183; doi:10.1371/journal.ppat.1003120)
Supplement: Table S4 — Statistics of the 20 lowest-energy conformer ensemble of MmpS452–140. (DOCX) [file ppat.1003120.s023.docx]

| **Number of Distance Constrains** |  |
| --- | --- |
| **NOE distance constraints** |  |
| intraresidue ( *i* – *j* = 0) | 293 |
| sequential ( *i* – *j* = 1) | 211 |
| medium-range ( 2≤ *i* – *j* ≤ 4) | 34 |
| long-range ( *i* – *j* ≥ 5) | 224 |
| Total | 762 |
| PRE constraints | 127 |
| **Number of Dihedral Angle Constraints** |  |
| *φ, ψ* | 63, 59 |
| **Average RMSD of Atomic Coordinates (Å) (52-131)** |  |
| backbone atoms | 0.54 |
| all heavy atoms | 1.27 |
| **Ramachandran plot analysis (%)** |  |
| most favorable region | 74.4 |
| additional allowed regions | 19.2 |
| generously allowed regions | 4.7 |
| disallowed regions | 1.8 |

**Table S4. Statistics of the 20 lowest-energy conformer ensemble of MmpS4_52-140_.**
