# Supplementary material for: Discovery of a Siderophore Export System Essential for Virulence of Mycobacterium tuberculosis
Source: PLoS Pathog. 2013 Jan 31;9(1):e1003120. doi: 10.1371/journal.ppat.1003120 (PMC3561183; doi:10.1371/journal.ppat.1003120)
Supplement: Text S1 — Supplementary materials and methods. Chemicals, enzymes, and DNA. Bacterial strains, media, and growth conditions. Plasmid construction. Construction of mutants in Mtb H37Rv and Mtb mc26230. Complementation of Mtb mutants. Preparation and analysis of protein extracts from M. tuberculosis. Southern blot analysis of M. tuberculosis. Protein overexpression, purification, and antibody production. Growth rescue experiments of M. tuberculosis mutants with iron utilization defects. Extraction and analysis of M. tuberculosis lipids. Subcellular fractionation of M. tuberculosis. Cloning, expression and purification of MmpS452–140. NMR spectroscopy. Assignment and data deposition. NMR Paramagnetic Relaxation Enhancement-Based Distance Measurements. Structure calculations. Interaction of MmpS4 and MmpL4. (DOCX) [file ppat.1003120.s024.docx]

**SUPPLEMENTARY MATERIALS AND METHODS**

**Chemicals, enzymes, and DNA.** Hygromycin B was purchased from Calbiochem. All other chemicals were purchased from Merck, Roche or Sigma at the highest purity available. Enzymes for DNA restriction and modification were purchased from New England Biolabs. Isolation and modification of DNA was performed as described(9). Oligonucleotides were obtained from Integrated DNA Technologies (Table S2).

**Bacterial strains, media, and growth conditions.** Unless mentioned otherwise, *Mtb* H37Rv strains were grown in Middlebrook 7H9 liquid medium supplemented with 0.2% glycerol, OADC (8.5 g/L NaCl, 20 g/L dextrose, 50 g/L bovine albumin (fraction V), 0.03 g/L catalase, 0.6 ml/L oleic acid), 0.02% Tyloxapol, or on Middlebrook 7H10 agar supplemented with 0.5% glycerol using premixed powders (Difco). Iron-free medium (7H9 or 7H10) was prepared according to the recipe of the manufacturer (Difco), except that ammonium ferric citrate and citric acid were omitted. Acid washed glassware was used in the preparation of iron-free media to avoid contamination with trace amounts of iron. Iron-free medium was solidified with Agar Noble (Difco). In the case of iron-deplete media, either the ferrous specific chelator, 2,2’-dipyridyl (DIP) or the ferric specific chelator desferrioxamine (DFO), were added at indicated concentrations. For the avirulent *Mtb* mc^2^6230 strains, media contained 0.2% casamino acids and 0.24 μg/ml pantothenate as supplements. *Escherichia coli* DH5α was used for all cloning experiments and was routinely grown in LB medium at 37°C. The following antibiotics were used when required: ampicillin (100 μg/ml for *E. coli*), kanamycin (30 μg/ml for mycobacteria), hygromycin (200 μg/ml for *E. coli*, 50 μg/ml for mycobacteria).

**Plasmid construction.** All plasmids utilized in this work are shown and briefly described in Table S3. The temperature-sensitive vectors pML1501 and pML1509 were used for deletion of *mmpS5* and *mmpS4*, respectively, in *Mtb*. Approximately 1000 bp up- and downstream of the genes targeted for deletion were amplified by PCR from chromosomal DNA using oligonucleotide pairs (Table S2) for *mmpS4* (1568/1569, 1570/1571; up- and downstream, respectively), and *mmpS5* (1409/1410, 1411/1412; up- and downstream, respectively). The restriction site SpeI was introduced into the upstream fragments while PacI and BfrBI were introduced into the downstream fragments. The *mmpS5* and *mmpS4* individual upstream sequences were digested with SpeI and ligated into the SpeI and SwaI (SwaI digestion yields blunt ends) digested temperature-sensitive replication vector pML523 (3) to yield pML1500 and pML1508, respectively. Subsequently, the *mmpS5* and *mmpS4* downstream sequences were digested with PacI and BfrBI, and introduced into similarly digested pML1500 and pML1508 such that up- and downstream regions flanked *loxP-gfp^2+^_m_-hyg-loxP* to yield the resulting plasmids pML1501 and pML1509, respectively. Construction of the *mmpS4L4* (pML1566) and *mmpS5L5* (pML1565) operon deletion vectors was accomplished in similar fashion as described above with the exception that the downstream homologous sequences were amplified using oligonucleotide pairs 1773/1774 and 1767/1768, respectively.

To construct the *mmpS4* and *mmpS5* L5 *attP* integrative expression plasmids pML1545 and pML1544 driven by their native promoters, *mmpS4* and *mmpS5* as well as their adjacent upstream ~500-600 bp promoter region were amplified by PCR from *Mtb* genomic DNA using the primer pairs 1811/1812 and 1813/1814, respectively. The sequences were digested with SpeI and HindIII, utilizing restriction sites that were introduced during amplification, and ligated into the similarly digested pML1342 (4), an integrative plasmid with L5 *attP*, to yield the *mmpS4* and *mmpS5* expression plasmids pML1545 and pML1544, respectively. In a similar fashion, the L5 *attP* integrative expression plasmid pML1802 containing *fxbA23_ gfp^2+^_m_* as an iron-dependent reporter was cloned by ligating the SpeI/HindIII digested insert from pML1801 (5) into similarly digested pML1342. To construct Ms6 *attP* integrative plasmids, the same *mmpS4* and *mmpS5* SpeI/HindIII digested amplified sequences from above were ligated into similarly digested pML2300 (4), an integrative plasmid with Ms6 *attP*, to yield the *mmpS4* and *mmpS5* expression plasmids pML1561 and pML1560, respectively. In order to construct pML1562, an empty vector Ms6 *attP* integrative plasmid, pML2300 was digested with SpeI and HindIII then subsequently incubated with Klenow fragment to fill in the 5’ overhangs to eliminate cohesive ends. This fragment was then self-ligated to create pML1562.

In order to construct the T7 promoter containing *E. coli* overexpression plasmids for MmpS4 and MmpS5, *E. coli* codon optimized *mmpS4* (*mmpS4_e_*) and *mmpS5* (*mmpS5_e_*) (GenScript) were digested with BamHI and HindIII. These fragments were ligated into the similarly digested pMAL-c5X (NEB) to yield the resulting plasmids pML1571 and pML1570. In order to delete the first 66 bp of *mmpS4_e_* and add an N-terminal 6xHis tag, *mmpS4_e_* was amplified by PCR using pML1571 as template using the primer pair 2356/1984. Similarly, to delete the first 78 bp of *mmpS5_e_* and add an N-terminal 6xHis tag, *mmpS5_e_* was amplified by PCR using pML1570 as template using the primer pair 2457/1984. The resulting PCR products were digested with NdeI and HindIII and ligated into similarly digested pET-28b(+) to yield the His-Mmps4_24-140_ and His-MmpS5_27-142_ overexpression plasmids pML1596 and pML1595, respectively.

**Construction of mutants in *Mtb* H37Rv and *Mtb* mc^2^6230.** To construct deletion mutants, the temperature-sensitive replication plasmids pML1501, pML1509, pML1565, pML1566, and pML1816, which contain homologous upstream and downstream regions of *mmpS5*, *mmpS4*, *mmpS5L5*, *mmpS4L4*, and *mbtD*, respectively, flanking the *loxP-gfp^2+^_m_-hyg-loxP* cassette, were transformed into *Mtb* H37Rv and mc^2^6230. In order to eliminate the potential for downstream polar effects, plasmids were designed so that upon unmarking of deletion mutants the remaining *loxP* would not contain any in frame stop codons. The plasmids were selected on 7H10/OADC/hyg plates at 37°C and single colonies were picked after 3-4 weeks of incubation and inoculated into 10 ml 7H9/OADC/hyg medium. This small 10 ml liquid culture was incubated at 37°C on a shaker for approximately 5 days until an OD_600_ of 1.0. Dilutions from 1 x 10^3^ to 1 x 10^6^ were plated on 7H10/OADC/hyg plates and incubated at 40°C to select for single-cross-over (SCO) events, while dilutions from 1 x 10^1^ and 1 x 10^2^ as well as undiluted culture were plated on 7H10/OADC/hyg plates supplemented with 2% sucrose and incubated at 40°C to select for double-cross-over (DCO) events. In the case of pML1816, the deletion plasmid for *mbtD*, the DCO selection plates also contained 10 μM hemin as an additional iron source to rescue the loss of siderophore biosynthesis in the event of DCO. After four weeks, SCO and DCO selection plates had numerous colonies. SCO and DCO candidates were screened for the presence of *xylE* and *gfp.* SCO candidates were both GFP and XylE positive while DCO candidates were GFP positive and XylE negative. SCO and DCO candidates were then picked into 10 ml of 7H9/OADC/hyg plates and incubated at 40°C for approximately 5 days to prepare chromosomal DNA, at which point correct candidates were confirmed by PCR analysis. For *mmpS4* and *mmpS5* single deletion mutants, correct DCO candidates were directly selected without the need of going through the SCO intermediate. The Cre recombinase expression vector pCreSacB1 (a kind gift from Dr. Adrie Steyn) was used to excise the *loxP*-flanked *gfp^2+^_m_-hyg* cassette from the chromosomes of the *mmpS4*, *mmpS5*, *mmpS4L4*, and *mmpS5L5* DCOs. The plasmid pCreSacB1 was transformed into the DCO strains and selected on 7H10/OADC/kan plates. After four weeks, colonies were screened for loss of *gfp* and transferred to 10 ml of 7H9/OADC medium and cultured at 37°C to an OD_600_ of 1.0. Whole cell PCR analysis was performed to confirm that the *loxP-gfp^2+^_m_-hyg-loxP* cassette was removed from the genomes. Then, in order to cure the unmarked *mmpS4* and *mmpS5* DCO strains of pCreSacB1, a series of 10-fold dilutions from single colonies having lost the *loxP-gfp^2+^_m_-hyg-loxP* cassette were plated on 7H10/OADC plates containing 2% sucrose and incubated at 37°C to counter-select against pCreSacB1. After three weeks, single colonies were streaked in parallel on 7H10/OADC, 7H10/OADC/kan, and 7H10/OADC/hyg plates to confirm the loss of the *hyg* and pCreSacB1. The unmarked *mmpS4* (Δ*mmpS4*::*loxP*) and *mmpS5* (Δ*mmpS5*::*loxP*) deletion mutants in *Mtb* H37Rv were named ML472 and ML405 (Table S1), respectively, while the deletion mutants in *Mtb* mc^2^6230 were named ML475 and ML406, respectively. The *mbtD* deletion mutants were not unmarked and retain the *loxP-gfp^2+^_m_-hyg-loxP* cassette. The *Mtb* mc^2^6230 *mbtD::hyg* deletion mutant ML1600 was constructed in a previous work (3) while the *Mtb* H37Rv *mbtD::hyg* deletion mutant was named ML1424. In order to construct the Δ*mmpS4/S5* double deletion mutants in *Mtb* H37Rv and mc^2^6230, the *mmpS4* deletion plasmid pML1509 was transformed into the Δ*mmpS5* single deletion mutants ML405 and ML406, respectively. The same steps as described above were used to construct the double deletion strain. The unmarked Δ*mmpS4/S5* double deletion strains (Δ*mmpS4*::*loxP*, Δ*mmpS5*::*loxP*) in *Mtb* H37Rv and mc^2^6230 were named ML482 and ML859, respectively. The triple mutants Δ*mmpS4/L4/S5* and Δ*mmpS4/S5/L5* were constructed by transforming the plasmids pML1566 and pML1565, respectively into the Δ*mmpS5* (ML405) and Δ*mmpS4* (ML475) avirulent parent strains. The unmarked Δ*mmpS4/S5/L5* was named ML1432 and the unmarked Δ*mmpS4/S5/L5* strain was named ML1433. All final deletion mutants were verified by Western and/or Southern blot analysis (Figs. S3, S4, S11). The triple deletion mutant Δ*mmpS4/S5*Δ*mbtD*::*hyg* in avirulent mc^2^6230 was constructed in the same fashion as ML1424, but using the unmarked double deletion mutant Δ*mmpS4/S5* (ML859) as the parent strain.

**Complementation of *Mtb* mutants.** In order to fully complement the Δ*mmpS4/S5* double deletion mutants in *Mtb* H37Rv and mc^2^6230, non-replicative integration plasmids utilizing the L5 and Ms6 mycobacterial phage integration systems were employed. By using two different phage integration systems it was possible to stably integrate *mmpS4* and *mmpS5* under the control of their native promoters at two different sites in the chromosome for full complementation. The L5 *attP* containing integrative plasmids pML1545 and pML1544 were used to singly complement the Δ*mmpS4/S5* double deletion mutant which enabled a direct head to head comparison of the effects of *mmpS4* and *mmpS5*, respectively, during infection studies. As control, the empty vector Ms6 *attP* containing integrative plasmid pML1562 was co-transformed in the singly complemented strains. The *mmpS4* singly complemented strains in *Mtb* H37Rv and mc^2^6230 were named ML620 and ML887, respectively. The *mmpS5* singly complemented strains in *Mtb* H37Rv and mc^2^6230 were named ML619 and ML886 (Table S1), respectively. For full complementation of the double deletion mutant, *mmpS4* was integrated at the L5 *attB* site using pML1545, while *mmpS5* was integrated at the Ms6 *attB* site using pML1560. The fully complemented strains in *Mtb* H37Rv and mc^2^6230 were named ML624 and ML889, respectively. The empty vector integrative plasmids pML1342 and pML1562 were co-transformed into *Mtb* H37Rv wt and mc^2^6230 wt resulting in strains ML617 and ML878, respectively, that were used as controls. Likewise, the double deletion mutants ML482 and ML859 were co-transformed with pML1342 and pML1562 to yield the strains ML618 and ML1401, respectively. Complementation was verified by Western blot analysis of protein extracts of the various strains (Fig. S4).

For IdeR reporter assays, the L5 *attP* integrative expression plasmid pML1802 containing *fxbA23_ gfp^2+^_m_* as an iron-dependent reporter was co-transformed into the avirulent double deletion mutant ML859 alongside either of the Ms6 *attP* integrative plasmds pML1561 and pML1560 containing *mmpS4* and *mmpS5* resulting in the strains ML892 and ML891, respectively. As control, both *Mtb* mc^2^6230 wt and ML859 were co-transformed with pML1802 and the empty integrative vector pML1562 to yield ML879 and ML890, respectively.

For genetic interaction studies between MmpS and MmpL proteins, complementation of the triple deletion mutants Δ*mmpS4/S5/L5* (ML1432) and Δ*mmpS4/S5/L5* (ML1433) was accomplished by using the L5 integrative plasmids pML1342 (empty vector), pML1544 (*mmpS5*) and pML1545 (*mmpS4*). For the Δ*mmpS4/S5/L5* strain this resulted in the strains ML1444 (empty), ML1445 (+*mmpS5*) and ML1446 (+*mmpS4*). For the Δ*mmpS4/S5/L5* this resulted in the strains ML1452 (empty), ML1437 (+*mmpS5*), and ML1438 (+*mmpS4*).

For co-transformations, 500 ng of each plasmid was mixed with 200 μl of competent cells and electroporated using standard settings. Cells were then immediately mixed with pre-warmed media and allowed to recover overnight after which they were plated on 7H10/OADC/hyg/kan plates and incubated at 37°C for 4-5 weeks. Single colonies were then picked and analyzed by whole cell PCR and Western blot for integration of both plasmids.

**Preparation and analysis of protein extracts from *M. tuberculosis*.** Protein extract preparation and analysis were performed as described in SI. Cultures of analyzed *Mtb* strains were allowed to grow until stationary phase (OD_600_ of 3). Ten ml of cell culture was harvested by centrifugation and washed one time with 2 ml of PBS (140 mM NaCl, 2 mM KCl, 10 mM K_2_HPO_4_/KH_2_PO_4_ pH 7.4) containing 1% SDS. Cells were then resuspended in PBS/1% SDS and transferred into a glass bead Lysing Matrix Tube (MP Biomedicals) and lysed in a FastPrep FP120 bead beater (BIO101/Savant) for two cycles (6,000 rpm for 45 sec). The samples were then heated while stirring using a Thermomixer R (Eppendorf) at 50°C and 800 rpm for 2 hours and centrifuged (12,000 rpm for 5 min). The supernatants were transferred to new tubes. After addition of protein loading buffer (160 mM Tris-HCl pH 7.0, 12% SDS, 32% glycerol, 0.4% bromophenol blue), samples were heated using the Thermomixer at 99°C for 20 min. Proteins were analyzed in Western blots using specific antibodies raised against RNA polymerase (RNAP) β subunit (Neoclone), MmpS4 (PA2915) and MmpS5 (PA2918) (this study). A horseradish peroxidase-coupled goat anti-mouse antibody (Sigma) and goat anti-rabbit antibody (Sigma) were used as secondary antibodies for RNAP and MmpS antibodies, respectively. Blots were developed using ECL Western blotting substrate (Pierce).

**Southern blot analysis of *M. tuberculosis*.** For southern blot analysis, 5 μg chromosomal DNA was isolated from deletion mutants, and digested with *Aat*I, *Apa*I, or *Nru*I, for analysis of the *mmpS4*, *mmpS5*, and *mbtD* genomic regions, respectively. For the *mmpS4L4 and mmpS5L5* genomic regions chromosomal DNA was digested with *Bam*HI and *Apa*I, respectively. Digested chromosomal DNA was separated on a 1% agarose gel and transferred in 10 x SSC (1.5 M NaCl, 0.15 M sodium citrate) to a positively charged nylon membrane (Amersham). The DNA was cross-linked to the membrane and prehybridized for 3 h at 42°C in Dig-Easy hybridization solution (Roche). For analysis of the *mmpS4*, *mmpS5*, and *mbtD* genomic regions, digoxigenin-labeled probes were generated by PCR from genomic DNA utilizing the primer pairs 1812/1569, 1814/1410, and 1953/1954 (Table S2), respectively. Analysis of the *mmpS4L4* and *mmpS5L5* genomic regions utilized the same probes that were used for *mmpS4* and *mmpS5*. Hybridization was carried out in the presence of 250 ng of digoxigenin-labeled PCR fragment at 42°C overnight. The membrane was washed twice for 5 min at room temperature with 2 x SSC, 0.1% SDS, and twice for 15 min at 68°C with 0.1 x SSC, 0.1% SDS. Detection of the hybridized digoxigenin-labelled probe was performed using a Dig nucleic acid detection kit (Roche).

**Protein overexpression, purification, and antibody production.** Competent cells of *E. coli* BL21 (DE3) were transformed with the plasmids pML1595 and pML1596, which contain a transcriptional fusion of codon usage-optimized *mmpS5_e_* and *mmpS4_e_* genes (GenScript), respectively, to the T7 promoter. The 5’ terminus of both *mmpS4* and *mmpS5* encodes a hydrophobic alpha helix that was deleted to achieve optimal overexpression of truncated MmpS4 and MmpS5. Additionally, a 6xHis encoding sequence was cloned at the beginning of the truncated *mmps4* and *mmpS5* to give N-terminal 6xHis tagged truncated MmpS4 (His-Mmps4_24-140_) and MmpS5 (His-MmpS5_27-142_) which aided in protein purification. The transformed cells were plated on LB agar supplemented with kanamycin (30 μg/ml). Single colonies were inoculated into 4 ml of LB medium with kanamycin. After overnight incubation at 37°C with shaking, 1 ml was used to inoculate 2 L flask containing 1 L of LB/0.3% glucose/kanamycin. Incubation was continued at 37°C with shaking until the optical density (OD) at 600 nm was 0.5. Then, isopropyl β-D-1-thiogalactopyranoside (IPTG) was added at a final concentration of 0.5 mM. After further incubation for 4 h, the cells were harvested by centrifugation (3,250 x g, 15 min), washed with PBS (140 mM NaCl, 2 mM KCl, 10 mM K_2_HPO_4_/KH_2_PO_4_ pH 7.4) containing 1 mM PMSF, and stored overnight at -20°C. Inclusion bodies were purified by first resuspending cells in PBS/1 mM PMSF (2 ml of buffer per 1 g of cells) and lysed by sonication (30 sec/ml of cell suspension, 12 Watt output power) on ice. The cell suspension was incubated with 1mg/ml lysozyme (Sigma) and 25 U Benzonase (Novagen) at 37°C for 2h with shaking. Unbroken cells and cellular debris was removed by centrifugation (4,000 x g, 15 min) at 4°C. The pellet was resuspended in 20 ml lysis buffer (50 mM Tris-HCl, 100 mM NaCl, 0.5% Triton-X 100, pH 8.0) and sonicated as above for 4 min, followed by two more cycles of centrifugation, resuspension, and sonication as described. Inclusion bodies were washed using 20 ml TS buffer (50 mM Tris-HCl, 100 mM NaCl, pH 8.0). Finally, inclusion bodies were collected by centrifugation (4,000 x g, 15 min, 4°C), and resuspended in 500 μl of TS buffer. The suspension of inclusion bodies was dissolved into TS buffer containing 8.5 M urea. In order to get rid of impurities, ion metal affinity chromatography (IMAC) using nickel resin was used to purify the His-Mmps4_24-140_ and His-MmpS5_27-142_ proteins.

Rabbit polyclonal antibodies were raised against IMAC-purified His-Mmps4_24-140_ and His-MmpS5_27-142_ at Open Biosystems (Huntsville, AL) using Titermax as adjuvant. The resulting antiserums were used for immunoblot analysis of purified protein and to verify protein levels of MmpS4 and MmpS5 of the various strains used in this study.

**Growth rescue experiments of *M. tuberculosis* mutants with iron utilization defects.** The avirulent wt *Mtb* mc^2^6230, Δ*mmpS4*, and Δ*mmpS5* strains were grown in 7H9 supplemented with 10% OADC, 0.2% casamino acids, 24 µg/ml pantothenate, 0.01% tyloxapol, and 0.1 mM dipyridyl (DIP) until they reached an OD_600_ of at least 2.0. Cells were harvested by centrifugation and the conditioned iron-deplete media was retained and sterilized by filtering through a 0.2 μm filter twice. The conditioned media was used to inoculate the Δ*mbtD::loxP* strain at an OD_600_ of 0.5. As controls, fresh 7H9 media as well as fresh media with 0.1 mM DIP were used for the growth of Δ*mbtD::loxP*. Optical densities were monitored at regular intervals for two weeks.

**Extraction and analysis of *M. tuberculosis* lipids.** The bacterial strains were grown in a static culture at 37°C in Sauton’s medium for four weeks so that surface pellicles could form. The strain ML618 was grown for eight weeks due to its delayed growth phenotype. After harvesting the pellicles the lipids were extracted according to (10) to provide apolar and polar lipid fractions. Briefly, the harvested pellicles were transferred into glass bottles and stirred in methanol/0.3% aqueous sodium chloride/petroleum ether (10:1:5 vol/vol) for 15 min. The mixture was allowed to separate. The upper petroleum ether layer was transferred into a new glass bottle and substituted with the same amount of petroleum ether. The cells were extracted for an additional 15 min before the upper petroleum ether layer was removed and combined with the first extract. This fraction contained the apolar lipids. The remaining aqueous phase was extracted with chloroform/methanol/0.3% aqueous sodium chloride (9:10:3 vol/vol) for 1h using a stirrer. The cellular debris was pelleted by centrifugation and the supernatant was transferred into a new glass bottle. The pellet was resuspended in chloroform/methanol/0.3% aqueous sodium chloride (5:10:4 vol/vol) and extracted for an additional 30 min. After centrifugation the supernatant was combined with the previous supernatant. The combined supernatants were mixed with chloroform/0.3% aqueous sodium chloride (1:1 vol/vol) for 5 min. Afterwards the mixture was separated by centrifugation and the lower phase was recovered. This fraction contained the polar lipids. The lipid containing fractions were dried and resuspended in chloroform according to the dry weight of the delipidated cells. The lipids were applied to 5 x 10 cm Macherey-Nagel glass plates for one-dimensional TLCs or to 10 x 10 cm Fluka silica gel aluminum-backed plates for two-dimensional TLCs and developed using several solvent systems. The lipids of the two-dimensional TLCs were visualized by spraying plates with 0.01% ethanolic Rhodamine G6 solution and enhanced with UV light. To visualize sugar-containing compounds, 0.2% anthrone in concentrated sulfuric acid and charring at 110^◦^C was used. The Dittmer-Lester reagent (Sigma) was used for revealing phosphorus-containing lipids. To visualize mycolic-acids, the TLC plates were sprayed with a 10 % copper sulfate in 8 % phosphoric acid solution and subsequently heated to 200^◦^C. Lipids were identified either by running purified lipids as references or by their R*_f_* value published previously (11-15).

The following solvent systems were used. System A: chloroform-methanol-water (60:35:8, vol/vol); B: chloroform/methanol/water (20:4:0.5 vol/vol); C: chloroform/methanol (95:5 vol/vol); D: 1^st^ petroleum ether (boiling point 60-80^◦^C)/ethyl acetate (98:2 vol/vol, three times), 2^nd^ petroleum ether/acetone (98:2 vol/vol); E: 1^st^ petroleum ether/acetone (92:8 vol/vol, three times), 2^nd^ toluene/acetone (95:5 vol/vol); F: 1^st^ chloroform/methanol/water (100:14:0.8 vol/vol), 2^nd^ chloroform/acetone/methanol/water (50:60:2.5:3 vol/vol).

**Subcellular fractionation of *M. tuberculosis.*** Experiment was carried out as described previously with some modifications (16). Briefly, avirulent *Mtb* ML878 (mc^2^6230 L5 *attB*::pML1342 (*loxP-xylE-int-hyg-loxP*); Ms6 *attB*::pML1562 (*loxP-xylE-int-kan-loxP*)) was grown until stationary phase (OD_600_ of 2) in 7H9 medium supplemented with 10% OADC, 0.02% Tyloxapol, 0.2% casamino acids, 0.24 µg/ml pantothenate and 0.1 mM dipyridyl. Cells were harvested by centrifugation and washed twice with PBS (140 mM NaCl, 2 mM KCl, 10 mM K_2_HPO_4_/KH_2_PO_4_ pH 7.4) containing 1 mM PMSF. Cell were resuspended in PBS/PMSF (4 ml of buffer per 1 g of cells) and lysed by sonication (20 min, 12 Watt output power). Cell suspension was incubated with 1mg/ml lysozyme (Sigma) and 50 U Benzonase (Novagen) at 37°C for 1h with shaking. Unbroken cells were removed by low speed centrifugation (3,200 x g for 10 min). The obtained supernatant (SN4) was diluted 5-fold and ultra-centrifuged at 135,000 x g for 1 h to separate cytosolic proteins (SN-100.1) from membrane fraction (P-100.1). P-100.1 was washed extensively to remove all cytosolic and membrane-attached proteins. SN-100.1 was centrifuged to remove all membrane proteins. All samples were mixed with protein loading buffer (160 mM Tris-HCl pH 7.0, 12% SDS, 32% glycerol, 0.4% bromophenol blue), boiled for 20 min and loaded on the 10% SDS-PAGE gel. The protein gel was blotted overnight at 50 mA in transfer buffer (25 mM Tris base, 192 mM Glycine, 0.1% SDS, 20% methanol) onto a polyvinylidene difluoride (PVDF) membrane (Amersham). After transfer proteins were detected using the rabbit antiserum raised against OmpAtb (17), IdeR (18), GlpX (Danilchanka et al, in preparation), Ag85 (Colorado State University), MmpS4 and MmpS5 (this study). A horseradish peroxidase-coupled goat anti-rabbit antibody (Sigma) was used as the secondary antibody. A horseradish peroxidase-coupled mouse antibody against HA (Sigma, #H6533) was used to detect HA-fusion proteins. Blots were developed using ECL Western blotting substrate (Pierce). LabWorks (UVP) chemoluminescence imaging system and software were used to visualize and quantify the luminescence.

**Cloning, expression and purification of MmpS4_52-140_.** The gene sequence of MmpS4_52-140_ was amplified from an *Mtb* H37Rv cDNA library and cloned into the pET-21b vector (Novagen) using standard cloning protocols. The recombinant plasmid was transformed into *E. coli* strain BL21(DE3)Gold (Stratagene). Uniformly ^15^N, ^13^C-labelled protein was over-expressed in M9 media supplemented with 3.0 g/L ^13^C D-glucose and 1.0 g/L ^15^NH_4_Cl (Cambridge Isotope Laboratories) as the sole carbon and nitrogen sources. The M9 culture was grown at 37^o^C until the OD_600_ reached 0.8, and was then induced with 0.8 mM IPTG (isopropyl-β-D-1-thiogalactopyranoside). After incubation at 25^o^C for 20 hours, the *E. coli* cells were harvested by centrifugation and resuspended in lysis buffer (70 mM Tris, 300 mM NaCl, pH 8.0). After sonication, cellular debris was removed by high-speed centrifugation. The 6×His-tagged protein was purified with Ni-NTA affinity chromatography (QIAGEN) following the manufacturer’s recommendations, and then further purified by size exclusion chromatography with a Superdex 75 10/300 GL column (GE Healthcare) to collect the monomer fraction (Fig. S16). Protein samples were analyzed by tricine-SDS-PAGE. The buffer was changed to 50 mM Na_2_HPO_4_/NaH_2_PO_4_, 2 mM DTT (dithiothreitol), pH 7.5.

**NMR spectroscopy.** The protein was dissolved in 500 µl NMR buffer (50 mM Na_2_HPO_4_/NaH_2_PO_4_, 2 mM DTT, pH 7.5, and 10% D_2_O). Solution NMR spectra were obtained at 298 K on Varian INOVA spectrometers operating at 500 or 700 MHz. A 2D ^1^H-^15^N HSQC (hetero-nuclear single quantum correlation spectroscopy) and some triple resonance experiments such as 3D CBCANH, 3D CBCACONH, 3D HNCO and 3D HNCA were acquired for backbone resonance assignments of the protein. Other experiments including 3D HCCH-TOCSY, 3D HCCH-COSY, 3D HCCONH and 3D HBHACONH were obtained for side chain resonance assignments. The NMR data were processed with NMRPipe (19, 20) and backbone and side chain resonances were assigned manually using NMRView software (21).

**Assignment and data deposition.** The ^1^H-^15^N HSQC spectrum of MmpS4_52-140_ is presented in Fig. S17, and the backbone amide assignments were illustrated. The well dispersed NMR peaks in the HSQC spectrum indicate that this protein is well structured. About 86% of the backbone resonances were assigned (73 out of 85 non-proline residues). In addition, 83 of 89 ^13^Cα, 72 of 82 ^13^Cβ, and 72 of 89 ^13^CO atoms have been assigned. Fig. S18 shows the assignment process of Cα and Cβ through HNCACB and CBCA(CO)NH spectra. The chemical shifts differences and the secondary structure of MmpS4_52-140_ evaluated by calculating the backbone torsion angels using TALOS+ (22) are shown in Fig S19, which suggest a structure consisting of β-strands.

**NMR Paramagnetic Relaxation Enhancement-Based Distance Measurements.** Paramagnetic relaxation enhancement (PRE) experiments were used to measure long range distance restraints. Select Ser residues were mutated to cysteine using PCR mutagenesis. Using the MmpS4_52-140_ plasmid, a total of three single-Cys mutants were prepared: S68C, S87C, and S99C. All mutations were selected to be located on relatively immobile sites. Prior to PRE measurements, ^1^H-^15^N HSQC experiments were performed to validate that these mutations did not perturb the native structure of MmpS4_52-140_. Single-Cys mutant forms of MmpS4_52-140_ were overexpressed and purified as described for wild type. Samples were reduced with DTT and then Cys-modified by the thiol-reactive nitroxide free radical probe, MTSL. Excess MTSL was later removed through desalting chromatography. For each spin-labeled single-cysteine mutant, a pair of 2D ^1^H-^15^N HSQC spectra were acquired for spin-labeled MmpS4_52-140_: one for the spin-labeled protein in the paramagnetic form, and one after adding adequate ascorbic acid to the sample in order to reduce the nitroxide, yielding the diamagnetic species. The distance restrains from PRE were obtained according to the method descried in the literature (23).

**Structure calculations.** Backbone ^13^Cα, ^13^Cβ, ^13^CO, and ^15^N chemical shifts were used to estimate backbone dihedral angles using the program TALOS+. Only restraints that were classified by this program as being in the highest confidence category were used in structural calculations. ^1^H-^1^H NOEs were obtained using the 3D ^15^N and ^13^C NOESY-HSQC experiment on uniformly-13C/15N double-labeled MmpS4_52-140_. The backbone dihedral angle, NOE, and long-distance restraints from PRE were used in the structure calculations for MmpS4_52-140_, using the program Xplor-NIH. The final 20 structures with the lowest energy were verified using PROCHECKNMR software.

**Interaction of MmpS4 and MmpL4.** The gene fragments encoding MmpL4_58-199_ and MmpL4_416-763_ were amplified from an *Mtb* H37Rv cDNA library and then inserted into the pET-28 vector with N-terminal 6×His-SUMO tags. The recombinant proteins were produced in *E. coli* BL21(DE3)Gold grown in LB media and induced with 0.8 mM IPTG at 37^o^C for 5h. The proteins were purified using Ni-NTA resin and subsequent size-exclusion chromatography (Superdex 75 10/300, GE Healthcare). SUMO protease was added to the purified proteins to cleavage the 6×His-SUMO tag. The tags were subsequently removed by Ni-NTA affinity chromatography. For pull down experiments, MmpS4_52-140_ with a C-terminal His-tag were first bound to Ni-NTA resin followed by a wash step to remove any unbound protein. Then, MmpL4_58-199_ or MmpL4_416-763_ were added and incubated for sufficient time. After washing away unbound protein, the protein complex was eluted and analyzed by tricine SDS-PAGE.

**SUPPLEMENTARY REFERENCES**

1. Sambrook, J., Fritsch, E. F. & Maniatis, T. (1989) *Molecular cloning: a laboratory manual* (Cold Spring Harbor Laboratory Press, Cold Spring Harbor, N. Y.).

2. Sambandamurthy, V. K., Derrick, S. C., Hsu, T., Chen, B., Larsen, M. H., Jalapathy, K. V., Chen, M., Kim, J., Porcelli, S. A., Chan, J., Morris, S. L. & Jacobs, W. R., Jr. (2006) *Vaccine* **24,** 6309-20.

3. Jones, C. M. & Niederweis, M. (2011) *J Bacteriol* **193,** 1767-70.

4. Huff, J., Czyz, A., Landick, R. & Niederweis, M. (2010) *Gene* **468,** 8-19.

5. Jones, C. M. & Niederweis, M. (2010) *J Bacteriol* **192,** 6411-7.

6. Guilhot, C., Gicquel, B. & Martin, C. (1992) *FEMS Microbiol Lett* **77,** 181-6.

7. Labidi, A., David, H. L. & Roulland-Dussoix, D. (1985) *Ann Inst Pasteur Microbiol* **136B,** 209-15.

8. Pelicic, V., Reyrat, J. M. & Gicquel, B. (1996) *J. Bacteriol.* **178,** 1197-1199.

9. Ausubel, F. A., Brent, R., Kingston, R. E., Moore, D. D., Seidmann, J. G., Smith, J. A. & Struhl, K. (1990) *Current protocols in molecular biology* (Greene Publishing and Wiley-Interscience, New York).

10. Tanya Parish, N. G. S. (2001) *Mycobacterium tuberculosis Protocols* (Humana Press, Totowa).

11. Sinsimer, D., Huet, G., Manca, C., Tsenova, L., Koo, M. S., Kurepina, N., kana, B., Mathema, B., Marras, S. A., Kreiswirth, B. N., Guilhot, C. & Kaplan, G. (2008) *Infect Immun* **76,** 3027-36.

12. Dandapat, P., Verma, R., Venkatesan, K., Sharma, V. D., Singh, H. B., Das, R. & Katoch, V. M. (1999) *Vet Microbiol* **65,** 145-51.

13. Tanya Parish, N. G. S. (1998) *Mycobacterium tuberculosis Protocols* (Humana Press.

14. Movahedzadeh, F., Wheeler, P. R., Dinadayala, P., Av-Gay, Y., Parish, T., Daffe, M. & Stoker, N. G. (2010) *BMC Microbiol* **10,** 50.

15. Deshayes, C., Bach, H., Euphrasie, D., Attarian, R., Coureuil, M., Sougakoff, W., Laval, F., Av-Gay, Y., Daffe, M., Etienne, G. & Reyrat, J. M. (2010) *Mol Microbiol* **78,** 989-1003.

16. Song, H., Sandie, R., Wang, Y., Andrade-Navarro, M. A. & Niederweis, M. (2008) *Tuberculosis* **88,** 526-44.

17. Senaratne, R. H., Mobasheri, H., Papavinasasundaram, K. G., Jenner, P., Lea, E. J. & Draper, P. (1998) *J. Bacteriol.* **180,** 3541-3547.

18. Dussurget, O., Rodriguez, M. & Smith, I. (1996) *Mol Microbiol* **22,** 535-44.

19. Delaglio, F., Grzesiek, S., Vuister, G. W., Zhu, G., Pfeifer, J. & Bax, A. (1995) *J Biomol NMR* **6,** 277-93.

20. Delaglio, F., Grzesiek, S., Vuister, G. W., Zhu, G., Pfeifer, J. & Bax, A. (1995) *Journal of Biomolecular NMR* **6,** 277-93.

21. Johnson, B. A. (2004) *Methods in Molecular Biology* **278,** 313-52.

22. Eid, J., Fehr, A., Gray, J., Luong, K., Lyle, J., Otto, G., Peluso, P., Rank, D., Baybayan, P., Bettman, B., Bibillo, A., Bjornson, K., Chaudhuri, B., Christians, F., Cicero, R., Clark, S., Dalal, R., Dewinter, A., Dixon, J., Foquet, M., Gaertner, A., Hardenbol, P., Heiner, C., Hester, K., Holden, D., Kearns, G., Kong, X., Kuse, R., Lacroix, Y., Lin, S., Lundquist, P., Ma, C., Marks, P., Maxham, M., Murphy, D., Park, I., Pham, T., Phillips, M., Roy, J., Sebra, R., Shen, G., Sorenson, J., Tomaney, A., Travers, K., Trulson, M., Vieceli, J., Wegener, J., Wu, D., Yang, A., Zaccarin, D., Zhao, P., Zhong, F., Korlach, J. & Turner, S. (2009) *Science* **323,** 133-8.

23. Battiste, J. L. & Wagner, G. (2000) *Biochemistry* **39,** 5355-65.
